# Nanoparticle-Mediated Lipid Metabolic Reprogramming of T Cells in Tumor Microenvironments for Immunometabolic Therapy

**DOI:** 10.1007/s40820-020-00555-6

**Published:** 2021-01-04

**Authors:** Dongyoon Kim, Yina Wu, Qiaoyun Li, Yu-Kyoung Oh

**Affiliations:** College of Pharmacy and Research Institute of Pharmaceutical Sciences, Seoul National University, Seoul, 08826 Republic of Korea

**Keywords:** Metabolic reprogramming, T cells, Fatty acid metabolism, Immunometabolic therapy, Mitochondrial function

## Abstract

**Highlights:**

- aCD3/F/AN, anti-CD3e f(ab′)2 fragment-modified and fenofibrate-encapsulated amphiphilic nanoparticle, reprogrammed mitochondrial lipid metabolism of T cells.
- aCD3/F/AN specifically activated T cells in glucose-deficient conditions mimicking tumor microenvironment, and exerted an effector killing effect against tumor cells.
- In vivo treatment with aCD3/F/AN increased T cell infiltration, cytokine production, and prevented tumor growth.

**Abstract:**

We report the activation of anticancer effector functions of T cells through nanoparticle-induced lipid metabolic reprogramming. Fenofibrate was encapsulated in amphiphilic polygamma glutamic acid-based nanoparticles (F/ANs), and the surfaces of F/ANs were modified with an anti-CD3e f(ab′)2 fragment, yielding aCD3/F/ANs. An in vitro study reveals enhanced delivery of aCD3/F/ANs to T cells compared with plain F/ANs. aCD3/F/AN-treated T cells exhibited clear mitochondrial cristae, a higher membrane potential, and a greater mitochondrial oxygen consumption rate under glucose-deficient conditions compared with T cells treated with other nanoparticle preparations. Peroxisome proliferator-activated receptor-α and downstream fatty acid metabolism-related genes are expressed to a greater extent in aCD3/F/AN-treated T cells. Activation of fatty acid metabolism by aCD3/F/ANs supports the proliferation of T cells in a glucose-deficient environment mimicking the tumor microenvironment. Real-time video recordings show that aCD3/F/AN-treated T cells exerted an effector killing effect against B16F10 melanoma cells. In vivo administration of aCD3/F/ANs can increase infiltration of T cells into tumor tissues. The treatment of tumor-bearing mice with aCD3/F/ANs enhances production of various cytokines in tumor tissues and prevented tumor growth. Our findings suggest the potential of nanotechnology-enabled reprogramming of lipid metabolism in T cells as a new modality of immunometabolic therapy.
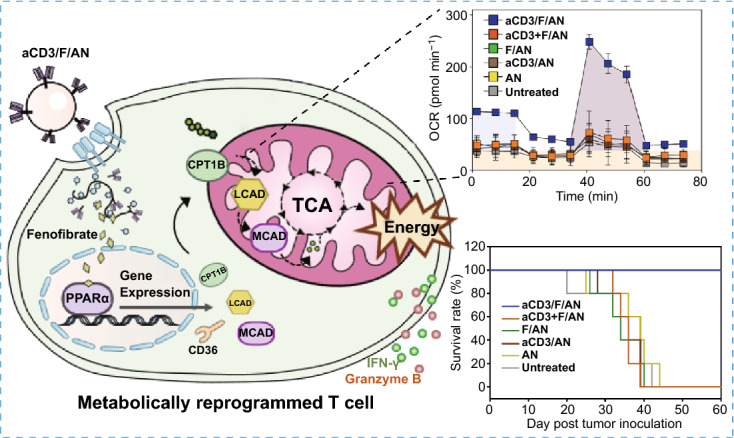

**Electronic supplementary material:**

The online version of this article (10.1007/s40820-020-00555-6) contains supplementary material, which is available to authorized users.

## Introduction

Anticancer chemicals and biologic necrosis factors have been delivered to tumors using various nanoparticles [1–4]. To date, the most studied anticancer nanomedicines for delivery of anticancer agents are those containing tumor-targeting ligands. Despite progress in nano-enabled targeted delivery of anticancer therapeutics to tumor tissues, clinical translation of these nanomedicines remains limited. One reason for the limited clinical translation of ligand-modified nanomedicines is the heterogeneity of tumors. The responsiveness of tumor cells to anticancer drugs can differ depending on tumor type. Moreover, the delivery of anticancer drugs to nonresponsive tumor cells can exert variable effects depending on the physiological properties of the cells [5, 6]. Even in drug-responsive tumor types, differing densities of target receptors on tumor cells can variably affect the extent of delivery of therapeutics to cancer cells [7]. Folate receptors and transferrin receptors have long been studied for targeted delivery of anticancer drugs to tumor cells. However, the density of these receptors on tumor cells varies such that delivery of nanoparticle anticancer drugs to tumor cells with a lower density of target receptors may be insufficient [8].

An emerging approach for circumventing this tumor cell heterogeneity focuses on leveraging tumor-adjacent immune cells in the tumor microenvironment [9, 10]. Compared with tumor cells, adjacent cells in the tumor microenvironments are reported to be less heterogeneous [11]. Reported targets in the microenvironment of various tumors include fibroblast activation protein, expressed on cancer-associated fibroblasts [12]; CD11c, expressed on dendritic cells [13]; and CD3, a biomarker of T cells whose expression on T cells is reported to be preserved in various tumors [14]. Exploiting this reduced heterogeneity of tumor-adjacent cells, we recently reported activation of cancer-associated fibroblasts for enhanced delivery of doxorubicin in graphene-based nanosheets [15]. It has been reported that dendritic cells in the tumor microenvironment can be activated by immune adjuvant-loaded polydopamine nanoparticles [16] and other nanoparticles, such as polymeric nanoparticles [17]. A number of studies addressing modulation of the tumor microenvironment have reported enhanced infiltration of T cells. However, the recruited T cells have been reported to lose their activity in the hypoglycemic tumor microenvironment [18]. Thus, there exists an unmet need for a therapeutic strategy for activating effector functions of T cells in the tumor microenvironment.

The importance of manipulating metabolic imbalances in immune cells in anticancer immunotherapy has become increasingly apparent [19]. Effector cytotoxic T cells have been reported to lose their activity because of the metabolically stressful conditions of the tumor microenvironment [20]. Glucose is known to be deficient in the tumor microenvironment, and the cytotoxic activity of T cells is impaired by a lack of energy. Thus, metabolic reprogramming of immune cells in the tumor microenvironment to allow utilization of different energy substrates would be a viable strategy for improving immunotherapy.

**Fig. 1:**
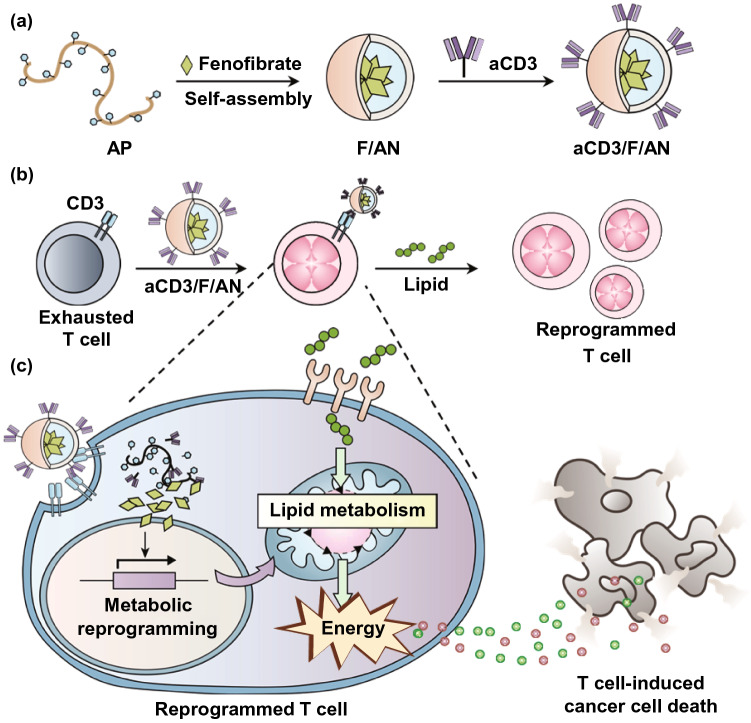
Metabolic reprogramming of T cells by aCD3/F/ANs. **a** Schematic illustration of the aCD3/F/AN preparation process. **b** Mechanism by which aCD3/F/ANs increase mitochondrial fatty acid metabolism in T cells. **c** Proposed effect of fatty acid metabolism reprogramming on T cell-mediated killing of cancer cells

In this study, we tested the hypothesis that metabolic reprogramming of T cells using a nanotechnology approach could activate T cells to fight against tumor cells (Fig. 1). To this end, we designed T cell-targeting nanoparticles encapsulating the lipid metabolism-activating drug molecule, fenofibrate, which is known to induce the expression of peroxisome proliferator-activated receptor (PPAR)-α and downstream fatty acid metabolism-related genes. It has been reported that promoting fatty acid metabolism by orally administered PPAR-α agonist can relieve metabolic stress from hypoglycemic condition in tumor microenvironment and preserve effector function of T cells, thereby enhancing anti PD-1 immunotherapy [21]. For T cell-targeted delivery, we entrapped fenofibrate in amphiphilic poly (γ-glutamic acid)-based nanoparticles (F/ANs) and further modified the nanoparticle surface with an anti-CD3e f(ab′)2 fragment, yielding aCD3/F/ANs. Here, we report that aCD3/F/ANs activate fatty acid metabolism and mitochondrial functions and stimulate the anticancer activity of T cells in tumor microenvironments.

## Experimental Section

### Synthesis of Amphiphilic Poly (γ-Glutamic Acid)

Amphiphilic poly (γ-glutamic acid) (AP) was synthesized by grafting phenylalanine ethyl ester with poly (γ-glutamic acid) (γ-PGA) through a carbodiimide crosslinking reaction, as described previously [22]. Briefly, 5.94 μmol of γ-PGA (50 kDa; BioLeaders, Daejeon, Republic of Korea) was dissolved in 30 mL of 0.3 M NaHCO_3_ solution followed by addition of 2.84 mmol of 1-(3-dimethylaminopropyl)-3-ethylcarbodiimide hydrochloride (EDC; Tokyo Chemical Industry Co., Ltd., Tokyo, Japan) with stirring on ice for 30 min. Next, 2.30 mmol of L-phenylalanine ethyl ester hydrochloride (Sigma-Aldrich, St. Louis, MO, USA) was added and the solution was stirred for an additional 24 h at 37 °C. For imaging and confocal microscopy, fluorescent dye-labeled AP (fAP) was synthesized by adding 3.2 μmol of fluoresceinyl glycine amide (5-(aminoacetamido) fluorescein) (Thermo-Fisher Scientific, Waltham, MA, USA) to a solution containing 2 μmol of γ-PGA, 0.96 mmol of EDC and 0.77 mmol of L-phenylalanine ethyl ester hydrochloride and allowing the reaction to proceed for 24 h. The resulting AP and fAP were purified by dialyzing first in triple distilled water for 48 h and then in methanol for 48 h using a Spectra/Pore dialysis membrane (MWCO, 3 kDa; Spectrum Labs, Rancho Dominguez, CA, USA), after which they were lyophilized and stored at 4 °C until use. The synthesis of AP was confirmed by ^1^H NMR spectroscopy.

### Preparation of Fenofibrate-Loaded and Anti-CD3 Antibody-Modified Nanoparticles

Fenofibrate was encapsulated in AP-based nanoparticles (ANs) or in fAP-based nanoparticles (fANs) using a thin film hydration method. In brief, 0.55 μmol of fenofibrate (Sigma-Aldrich) in 1 mL methanol was added into 1 mL of 0.1 mM AP or fAP in methanol solution. After solvent removal using a rotary evaporator (CCA-1110; Tokyo Rikakikai, Tokyo, Japan), the resulting film was hydrated with 1 mL of phosphate-buffered saline (PBS; pH 7.4) and sonicated for 10 min. The resulting F/ANs or fenofibrate-loaded fANs (F/ANs) were purified from unencapsulated fenofibrate by centrifugation at 12,000×g for 3 min. For modification with anti-CD3 antibody, F/ANs (3.5 mg) or F/fANs (3.5 mg) were first mixed with 6.4 μmole of EDC, then reacted on ice for 5 min and allowed to stand at room temperature (RT) for an additional 5 min. A solution of anti-mouse CD3e f(ab′)2 antibody (Bio X Cell, West Lebanon, NH, USA, catalog #BE0001-1FAB, lot #659618A1) was added to the EDC-activated F/AN suspension at a 1:50 molar ratio of antibody to AP and stirred at RT for 6 h. Free antibody was removed by dialysis (MWCO, 300 kDa; Spectrum Labs), and after quantifying the amount of antibody on the nanoparticle surface by BCA assay (Thermo-Fisher Scientific), aCD3/F/ANs or anti-mouse CD3e f(ab′)2 antibody-conjugated F/fANs (aCD3/F/fANs) were concentrated from reactants using an Amicon filter (Merck, Kenilworth, NJ, USA). In some experiments, anti-mouse CD3e f(ab′)2 antibody was conjugated to plain ANs (aCD3/ANs) or to fANs (aCD3/fANs). Physical mixture of equivalent amount of anti-mouse CD3e f(ab′)2 antibody with F/ANs (aCD3 + F/ANs) or with F/fANs (aCD3 + F/fANs) were used as control groups.

### Quantification of Encapsulated Fenofibrate

The amount of fenofibrate encapsulated in ANs was analyzed by high-performance liquid chromatography (HPLC) using a Hewlett Packard model 1100 system (Hewlett Packard, Palo Alto, CA, USA) equipped with a reverse-phase C18 HPLC column (Nucleosil 100-5 C18; Macherey–Nagel, Düren, Germany). Lyophilized nanoparticles were dissolved in 200 μL of MeOH and injected into the C18 column. The column (temperature, 40 °C) was eluted using a mobile phase consisting of acetonitrile:water (pH of water adjusted to 4 with acetic acid) (90:10, v/v) at a flow rate of 1 ml/min. The peak of fenofibrate was monitored by measuring absorbance at 287 nm using at UV detector. Fenofibrate concentration was determined by reference to a standard curve prepared using a series of fenofibrate solutions in methanol.

### Characterization and Stability Studies

Nanoparticles were characterized by size, surface charge, morphology, and elemental composition. The size distribution and zeta potential of nanoparticles were measured by dynamic light scattering (DLS) using an ELSZ-1000 instrument (Otsuka Electronics Co., Osaka, Japan). The morphology of nanoparticles was visualized by transmission electron microscopy (TEM) using a JEM1010 transmission electron microscope (JEOL, Tokyo, Japan). The presence of elemental oxygen, chlorine and sulfur in nanoparticles was determined by energy-dispersive X-ray spectroscopy (EDS-STEM) using a JEM-2100 F transmission electron microscope (JEOL). The stability of nanoparticles was monitored for up to 7 days. F/ANs and aCD3/F/ANs were stored in PBS at 4 °C. The sizes of particles were measured daily by DLS using an ELSZ-1000 instrument (Otsuka Electronics Co.).

### In Vitro Study of pH-Dependent Release

The pH dependence of fenofibrate release from ANs was tested using a dialysis approach. Fenofibrate released from ANs was quantified by immersing a dialysis bag (MWCO, 3.5 kDa; Spectrum Labs) containing aCD3/F/ANs in 15 mL PBS containing 0.1% (w/v) Tween-80 (Sigma-Aldrich) at different pH values. At each time point, 1 mL of dialysis buffer was collected and the volume of dialysis was maintained by adding fresh buffer. Collected samples were lyophilized and dissolved in 200 μL methanol, and the amount of fenofibrate release was analyzed by HPLC.

### Animals

Five-week-old C57BL/6 mice were purchased from Raon Bio and maintained under standard pathogen-free conditions at the Animal Center for Pharmaceutical Research, Seoul National University. All animal experiments were performed according to the Guidelines for the Care and Use of Laboratory Animals of the Institute of Laboratory Animal Resources, Seoul National University (approval number, SNU-190417-15(E)).

### Evaluation of CD3 Expression

The expression levels of CD3 were determined at various cells including murine lung fibroblast cell line MLg (Korean Cell Line Bank, Seoul, Republic of Korea), human lung fibroblast cell line MRC-5 (Korean Cell Line Bank), human embryonic kidney cell line 293 T (ATCC, Manassas, VA, USA), murine bone marrow-derived macrophages (BMDM), murine bone marrow-derived dendritic cells (BMDC), and T cells. BMDM and BMDC were differentiated from monocytes as previously described [16, 23]. T cells were isolated from spleens of C57BL/6 mice using nylon wool columns [24]. MLg, MRC-5, 293 T, BMDM, BMDC, and T cells were stained with FITC-conjugated anti-mouse CD3 antibody (BioLegend, San Diego, CA, USA; catalog #100203, lot #B263028) for 1 h. The expression of CD3 was evaluated by flow cytometry.

### In Vitro Study of Nanoparticle Uptake

In vitro cellular uptake of nanoparticles was evaluated using flow cytometry and fluorescence microscopy. For flow cytometry, MLg, MRC-5, 293 T, BMDM, BMDC and T cells were seeded onto 24-well plates (SPL Life Sciences) at 1 × 10^6^ cells/well and treated with 2 mg mL^−1^ of fluorescein isothiocyanate (FITC)-labeled various nanoparticles for 4 h. Cells were harvested, washed with PBS, and analyzed by flow cytometry. For fluorescence microscopy, T cells were seeded onto 24-well plates (1 × 10^6^ cells/well) and treated with 2 mg mL^−1^ FITC-labeled nanoparticles for 4 h. After washing and resuspending cells in 100 μL PBS, cell suspensions were plated onto poly-L-lysine coverslips (Corning, New York, NY, USA) and allowed to completely attach (30 min). Cells were then fixed with 4% formaldehyde in PBS for 10 min, washed with PBS, and stained with DAPI (4′,6-diamidino-2-phenylindole, Sigma-Aldrich). The fluorescence of cells was observed using a confocal laser-scanning microscope (LSM 5 Exciter; Carl Zeiss, Inc., Jena, Germany).

### In Vitro Cell Viability Study

In vitro viability of cells was measured after treatment of nanoparticles. After treatment with various nanoparticles, T cells were suspended in RPMI medium containing 10% WST-1 reagent. After incubating for 2 h at 37 °C, absorbance of the medium at 430 nm was measured using a Multi-Reader (Molecular Devices, San Jose, CA, USA). The other adherent cells including MLg, MRC-5, 293 T, BMDM and BMDC were incubated with culture medium containing 10% MTT reagent. After incubating for 2 h at 37 °C, absorbance of medium at 570 nm was detected using a Multi-Reader (Molecular Devices).

### In Vitro Study of PPARα, CD36 and Fatty Acid Oxidation-Associated Gene Expression

Expression levels of PPARα, CD36 and fatty acid oxidation-associated genes in T cells following treatment with nanoparticles were measured by reverse transcription-polymerase chain reaction (RT-PCR), flow cytometry and western blot. The fatty acid oxidation-associated genes and gene products measured included carnitine palmitoyltransferase 1B (CPT1B), acyl-CoA dehydrogenase medium chain (MCAD) and acyl-CoA dehydrogenase long chain (LCAD) [21, 25]. Isolated mouse T cells were seeded onto 24-well plates (1 × 10^6^ cells/well) and treated with various nanoparticle preparations containing 20 μM fenofibrate for 24 h. For RT-PCR, total RNA was isolated from T cells using the TRIzol reagent (Invitrogen, Carlsbad, CA, USA). cDNA was synthesized from mRNA using an AccuPower RT PreMix (Bioneer, Daejeon, Republic of Korea). Quantitative real-time RT-PCR was performed using a LightCycler FastStart DNA Master SYBR Green I system (Roche, Basel, Switzerland). Primer sequences used in RT-PCR are listed in Table S1. For western blot, T cells were lysed with a commercial radioimmunoprecipitation assay lysis buffer (Rockland, Limerick, PA, USA) containing a cocktail of protease inhibitors (cOmplete™, Mini, EDTA-free Protease Inhibitor Cocktail, Sigma-Aldrich (Roche). The amounts of protein were quantified by BCA assay and boiled at 95 °C 10 min. As primary antibodies, anti-mouse CPT1B (Proteintech, Rosemont, IL, USA; catalog #22170–1-AP, lot #00048665), anti-mouse LCAD (Proteintech, catalog #17526-1-AP, lot #00051417), anti-mouse MCAD (Proteintech, catalog #55210-1-AP, lot #09000327) and anti-mouse β-actin (Cell signaling, Denver, MA, USA; catalog #8457, lot #6) were used. Horseradish peroxidase-linked anti-rabbit IgG (Cell signaling, catalog #7074, lot #28) was used as secondary antibody. Amersham ECL Prime Western Blotting detection reagent (GE Healthcare, Chicago, IL, USA) was used for signal detection.

Expression levels of PPARα protein were assessed by flow cytometry. Briefly, T cells were seeded onto 24-well plates (1 × 10^6^ cells/well) and treated with various nanoparticle preparations containing 20 μM fenofibrate for 48 h. Cells were harvested, washed with PBS, and stained with FITC-conjugated anti-mouse CD3 antibody (BioLegend) for 1 h. Cells were fixed and permeabilized using a transcription factor buffer set (BioLegend; catalog #424401) and incubated with Alexa Fluor 647-conjugated anti-mouse PPARα antibody (Santa Cruz Biotechnology, Santa Cruz, CA, USA; catalog #sc-398394, lot #H1017) for 1 h.

Protein expression levels of the fatty acid translocase, CD36, were analyzed by flow cytometry. Briefly, T cells seeded onto a 24-well plate (1 × 10^6^ cells/well) were treated with various nanoparticle preparations containing 20 μM fenofibrate for 48 h and then stained with FITC-conjugated anti-mouse CD3 antibody and allophycocyanin (APC)-conjugated anti-mouse CD36 antibody (BioLegend; catalog #102611, lot #B270594) for 1 h.

### Assessment of Mitochondrial Activation

Mitochondrial activation was evaluated based on morphology, membrane potential and oxygen consumption rate (OCR). T cells were seeded onto a 24-well plate (1 × 10^6^ cells/well) and stimulated with anti-CD3/CD28 antibody-tethered Dynabeads (Thermo-Fisher Scientific) at a 1:1 ratio of T cells to beads in the presence of interleukin (IL)-2 (10 ng mL^−1^) for 48 h. After the removal of beads, T cells were treated with various nanoparticle preparations containing 20 μM fenofibrate in the presence of IL-2 (10 ng mL^−1^) for 48 h. Cells were then starved by incubating for 24 h in low-glucose medium (XF DMEM Base Medium) containing 0.5 mM XF glucose, 1 mM XF glutamine, 0.5 mM carnitine (Sigma-Aldrich), 1% fetal bovine serum (FBS) and 40 μM palmitate-bovine serum albumin (BSA; Seahorse Biosciences, Billerica, MA, USA). For TEM observation of mitochondrial morphology, T cells were fixed with Karnovsky’s solution and pelleted, after which cell pellets were fixed with 1% osmium tetroxide for 1 h and stained with 0.5% uranyl acetate overnight. Cell pellets were dehydrated with ethanol and sequentially immersed in ethanol/Spurr’s resin (1:1) solution and ethanol/Spurr’s (1:2) solution for 1 h each [26]. Cells were then incubated with Spurr’s resin again at 4 °C overnight, followed by incubation at 70 °C overnight. Thin sections (60 nm) were prepared and observed by TEM (Talos L120C; Thermo Fisher Scientific, Inc.).

Mitochondrial membrane potential was measured by flow cytometry and confocal microscopy. For flow cytometry, cells were incubated with 0.125 μM MitoTracker Orange CMTMRos (Life Technologies) for 30 min and analyzed by flow cytometry. For confocal microscopy, cells were seeded onto poly-L-lysine coverslips after staining with MitoTracker. Nuclei were counterstained with DAPI and fixed with 4% formaldehyde. The fluorescence of T cells was observed by confocal laser-scanning microscopy (TCS8; Leica Microsystems GmbH, Wetzlar, Germany).

OCR and extracellular acidification rate (ECAR) were measured with an XFp Extracellular Flux analyzer (Seahorse Biosciences) using an XFp Cell Mito Stress Test Kit (Seahorse Biosciences) and XFp Glycolysis Stress Kit (Seahorse Biosciences), according to the manufacturer’s instructions. For OCR measurements, T cells were seeded onto a Cell-Tak (Seahorse Biosciences) pre-coated XFp culture microplate (4 × 10^5^ cells/well). Immediately prior to OCR measurements, 100 μM of palmitate-BSA was added to the plate. During measurements, 2 μM oligomycin, 1.5 μM carbonyl cyanide-4 (trifluoromethoxy)phenyl hydrazone and 1 μM rotenone and antimycin A mixture were added to each well. For ECAR measurements, T cells were seeded onto a Cell-Tak pre-coated XFp microplate (4 × 10^5^ cells/well). During the measurement, 10 mM glucose, 1 μM oligomycin and 100 mM 2-deoxy-D-glucose were added to each well.

### In Vitro T Cell Proliferation Test

The effect of nanoparticles on T cell proliferation was studied using cell apoptosis, cell proliferation, and live/dead cell assays. Mouse T cells were seeded onto a 24-well plate (1 × 10^6^ cells/well) and activated by adding anti-CD3/CD28 Dynabeads at a T cell:Dynabeads ratio of 1:1 in the presence of IL-2 (10 ng mL^−1^) for 48 h. After removing Dynabeads, T cells were treated with various nanoparticle preparations containing 20 µM fenofibrate for 48 h in the presence of IL-2 (10 ng mL^−1^). The medium was then changed to low-glucose medium supplemented with 40 μM palmitate-BSA and incubated for an additional 24 h.

Apoptosis of T cells was measured using an Annexin V Apoptosis Detection Kit FITC (Thermo-Fisher Scientific). Apoptotic cells were detected by staining cells with annexin V and propidium iodide (PI). Cell viability was measured using WST-1 reagent (Sigma-Aldrich). In brief, T cells were suspended in 100 μL RPMI medium containing 10% WST-1 reagent and then seeded onto a 96-well plate. After incubating for 2 h at 37 °C, absorbance of the medium at 430 nm was measured using a Multi-Reader (Molecular Devices). For live/dead assays, T cells were harvested and stained with 2 μM calcein AM and 4 μM PI for 15 min. Cell viability was assessed by examining under a fluorescence microscope (DM IL; Leica).

### In Vitro Measurement of Lipid Uptake by T Cells

Lipid uptake by T cells was measured by flow cytometry using fluorescently labeled lipids. T cells were seeded onto a 24-well plate (1 × 10^6^ cells/well) and stimulated with anti-CD3/CD28 antibody-tethered Dynabeads at a T cell to bead ratio of 1:1 in the presence of IL-2 (10 ng mL^−1^) for 48 h. Dynabeads were then removed, and T cells were further treated with various nanoparticle preparations containing 20 μM fenofibrate in the presence of IL-2 (10 ng mL^−1^) for 48 h. The medium was then replaced with fresh RPMI-1640 medium, and T cells were treated with 1 µM 4,4-difluoro-5,7-dimethyl-4-bora-3a,4a-diaza-s-indacene-3-hexadecanoic acid (BODIPY C_16_, Thermo-Fisher Scientific), a fluorescent fatty acid, at 37 °C for an additional 2 h. T cells were harvested and washed with PBS, and lipid uptake was analyzed by flow cytometry.

### In Vitro Measurement of T Cell Secretion of Fatty Acid Metabolites

Secretion of fatty acid metabolites from T cells was evaluated by assaying β-hydroxybutyrate in culture medium. Briefly, isolated T cells were seeded in a 24-well plate (1 × 10^6^ cells/well) and activated with anti-CD3/CD28 antibody-tethered Dynabeads at a T cell to bead ratio of 1:1 in the presence of IL-2 (10 ng mL^−1^) for 48 h. Dynabeads were removed, and T cells were treated with nanoparticle preparations containing 20 μM fenofibrate. After incubation with nanoparticles for an additional 48 h, cells were washed with PBS and resuspended in low-glucose medium with 40 μM palmitate-BSA. After starvation for 24 h, β-hydroxybutyrate concentration in the culture medium was measured using a β-Hydroxybutyrate Assay Kit (Abcam, Cambridge, England).

### In Vitro Assessment of the Cancer Cell-Killing Activity of Nanoparticle-Stimulated T Cells

The ability of nanoparticle-treated T cells to kill B16F10 melanoma cells was measured by fluorescence dye staining and live, real-time video monitoring. T cells from splenocytes were exposed to tumor-associated antigens and treated with various nanoparticle preparations. To better mimic the in vivo tumor microenvironment, we isolated splenocytes from spleens of adjuvant-treated B16F10 tumor-bearing mice. In brief, C57BL/6 mice were inoculated subcutaneously with B16F10 cells (5 × 10^5^ cells). Seven days after tumor inoculation, doxorubicin (DOX; Sigma-Aldrich), used as an immunogenic cell death inducer [27], was injected intratumorally at a dose of 50 μg/mouse together with monophosphoryl lipid A (MPL; InvivoGen, San Diego, CA, USA), used as an adjuvant [28], at a dose of 20 μg/mouse [29]. After 2 days, mice were sacrificed and splenocytes were isolated from the spleen and antigenically stimulated with B16F10 lysates, prepared by repeatedly freezing and thawing (5 cycles) B16F10 cells (2 × 10^5^ cells mL^−1^). Splenocytes were incubated with the resulting tumor lysates for 48 h, after which collected T cells were treated with various nanoparticle preparations with or without 20 µM fenofibrate for 48 h, and then further treated with low-glucose medium containing 40 µM palmitate-BSA for 24 h.

The cancer cell-killing activity of T cells was evaluated by first seeding B16F10 cells, pre-stained with CellTracker Green CMFDA Dye (Thermo-Fisher Scientific), onto a 24-well plate (1 × 10^4^ cells/well) and co-culturing them with B16F10 tumor lysate-stimulated T cells (1 × 10^6^ cells/well). After 24 h, cancer cells were stained with PI, and the population of dead (CMFDA^+^/PI^+^) cancer cells was analyzed by flow cytometry. In some experiments, the anticancer effects of T cells were evaluated by staining T cells with FITC-conjugated anti-mouse CD3 antibody or APC-conjugated anti-mouse CD3 antibody (BioLegend; catalog #100235, clone 17A2, lot #B256435). Then, the cells were fixed and permeabilized using a transcription factor buffer set (BioLegend) and incubated with Alexa Fluor 647-conjugated anti-mouse granzyme B antibody (BioLegend; catalog #515406, clone GB11, lot #B233111) or PE-conjugated anti-mouse IFN-γ antibody (BioLegend; catalog #505808, clone XMG1.2, lot #B265789). After 1 h, the percentages of CD3^+^Granzyme B^+^ or CD3^+^IFN-γ^+^cells were analyzed by flow cytometry. For real-time monitoring of stimulated T cell-mediated cancer cell lysis, T cells pre-stained with CellTracker Green CMFDA dye were seeded onto a 24-well plate and cocultured with B16F10 cells stained with CellTracker Red CMTPX dye (Thermo-Fisher Scientific) at a T cell:cancer cell ratio of 100:1. Cancer cell lysis was recorded in real time over 45 h using an Operetta High-Content Imaging System (PerkinElmer, Waltham, MA, USA).

### In Vitro Study of FoxP3 Expression

The expression levels of FoxP3 in T cells after treatment with various nanoparticles were evaluated by flow cytometry. T cells were seeded onto 24-well plates (1 × 10^6^ cells/well) and treated with various nanoparticles containing 20 μM fenofibrate for 48 h. Cells were harvested, washed with PBS and stained with FITC-conjugated anti-mouse CD3 antibody, PE/Cy5-conjugated anti-mouse CD25 antibody (Biolegend; catalog #102010, lot #B277468) and PE-conjugated anti-mouse CD4 antibody (Biolegend; catalog #116006, lot #B255181) for 1 h. Cells were fixed and permeabilized using a transcription factor buffer (BioLegend) and incubated with APC-conjugated anti-mouse FoxP3 antibody (Invitrogen; catalog #17-5773-82, lot #1984797) for 1 h.

### In Vivo T Cell Targeting of Nanoparticles

In vivo T cell targeting of nanoparticles was evaluated using molecular imaging and flow cytometry. Six-week-old mice were subcutaneously inoculated in the right flank with 5 × 10^5^ B16F10 cells. After 7 days, mice were injected intratumorally with various FITC-labeled nanoparticle preparations. For molecular imaging, the fluorescence intensity of tumor tissue was recorded at various time points using an IVIS Spectrum in Vivo Imaging System (PerkinElmer). For flow cytometry, tumors were extracted at 24 h immediately after injection of FITC-labeled nanoparticles. T cells in extracted tumors were analyzed by incubation with APC-conjugated anti-mouse CD3 antibody and the percentage of CD3^+^FITC^+^ cells was determined by flow cytometry.

The distribution of nanoparticles to T cells was visualized by first extracting the tumor and fixing it by incubating with 4% formaldehyde for 4 h at RT. The tissue was then embedded in OCT cryostat sectioning medium, frozen at −80 °C and sectioned at 10 μm using a Leica CM 3050 S microtome (Leica). The tumor sections were washed three times with PBS and blocked with 1% BSA in PBS for 30 min. After blocking, the sections were incubated with APC-conjugated anti-CD3 antibody (BioLegend; catalog #100235, lot #B290370) for 12 h at 4 °C. After staining with DAPI, tissue fluorescence was assessed under a confocal laser-scanning microscope (LSM 5 Exciter; Carl Zeiss).

### In Vivo PPARα Expression Measurement

T cell lipid metabolism in vivo was evaluated by measuring PPARα expression. B16F10 cells (5 × 10^5^ cells) were subcutaneously inoculated into the right flank of 5-wk-old C57BL/6 mice. After 7 days, DOX (50 μg/mouse) and MPL (20 μg/mouse) were injected intratumorally. On days 10 and 12 after B16F10 cell inoculation, various nanoparticle preparations (18 μg fenofibrate/mouse) were administered intratumorally. On day 14, mice were sacrificed and extracted tumors were stained with FITC-conjugated anti-mouse CD3 antibody and Alexa Fluor 647-conjugated anti-mouse PPARα antibody (Santa Cruz Biotechnology; catalog #sc-398394, lot #H1017) for 1 h, followed by flow cytometry. For immunofluorescence staining, tumors were extracted and fixed with 4% formaldehyde for 4 h at RT, after which the tissue was embedded in OCT and cryosectioned at a thickness of 10 μm with a Leica CM 3050 S microtome (Leica). After permeabilizing tissue with 0.3% Triton X-100 (Sigma-Aldrich) in PBS for 10 min and blocking with 0.025% Triton X-100 and 1% BSA in PBS for 1 h, the sections were incubated with FITC-conjugated anti-mouse CD3 antibody and Alexa Fluor 647-conjugated anti-mouse PPARα antibody (Santa Cruz Biotechnology). Tissues were then counterstained with DAPI and observed under a confocal laser-scanning microscope (LSM 5 Exciter; Carl Zeiss).

### In Vivo Lipid Uptake and Metabolite Imaging in T Cells

Lipid uptake and metabolism in T cells were assessed by flow cytometry and matrix-assisted laser desorption ionization (MALDI) imaging mass spectrometry, respectively. Briefly, mice were injected intratumorally with 25 μg kg^−1^ BODIPY C_16_ on the day after the last injection of nanoparticles. After 4 h, tumors were extracted and T cells were stained with APC-conjugated anti-mouse CD3 antibody for 1 h and the percentage of CD3^+^BODIPY^+^ T cells was analyzed by flow cytometry. For lipid metabolite analysis, F/ANs and aCD3/F/ANs were administered intratumorally on days 10 and 12. On day 14, tumors were extracted and embedded in 2% carboxymethyl cellulose (Sigma-Aldrich) and cryosectioned at 10 μm with a Leica CM 3050 S microtome (Leica). Frozen tissue sections were mounted onto indium-tin-oxide–coated glass slides (Bruker, Billerica, MA, USA) and then were sprayed with 10 mg/ml solution of a α-cyano-4-hydroxycinnamic acid (Bruker) in 70% acetonitrile with 0.1% trifluoroacetic acid. The samples were scanned over the relevant molecular weights, and the distribution of representative metabolites was determined using a rapifleX system (Bruker).

### In Vivo Assessment of Fatty Acid Oxidation-Associated Gene Expression

In vivo expression levels of fatty acid oxidation-associated genes in CD3-positive T cells were measured RT-PCR and western blot. Briefly, tumors were extracted and digested by 1 mg mL^−1^ collagenase (Sigma-Aldrich). Single cells were stained with APC conjugated anti-mouse CD3 antibody and CD3-positive cells were collected using BD FACSAria™ III sorter (BD Biosciences, San Jose, CA, USA). For RT-PCR, total RNA was isolated from CD3-expressing T cells using the TRIzol reagent (Thermo-Fisher Scientific). cDNA was synthesized from mRNA using an AccuPower RT PreMix. Quantitative real-time RT-PCR was performed using a LightCycler FastStart DNA Master SYBR Green I system. For western blot, CD3-expressing T cells were lysated with RIPA buffer containing protease inhibitor. The amounts of protein were quantified by BCA assay. Primary antibodies for detecting following proteins were uses: anti-mouse CPT1B (Proteintech), anti-mouse LCAD (Proteintech), anti-mouse MCAD (Proteintech) and anti-mouse β-actin (Cell signaling). Horseradish peroxidase-linked anti-rabbit IgG (Cell signaling) was used as secondary antibody. Amersham ECL Prime Western Blotting detection reagent was used for signal detection.

### In Vivo Antitumor Efficacy

The in vivo antitumor activity of various nanoparticle preparations was evaluated by monitoring B16F10 tumor growth. B16F10 cells (5 × 10^5^ cells) were subcutaneously inoculated into the right flank of 5-week-old C57BL/6 mice. After 7 d, DOX (50 μg/mouse) and MPL (20 μg/mouse) were injected intratumorally, and on days 10 and 12, various nanoparticle preparations containing fenofibrate (18 μg/mouse) were administered intratumorally. Tumor size was measured in two dimensions every 2 days using a slide caliper, and tumor volume was calculated as *a* × *b* × *b* × 0.5, where *a* and *b* are the lengths of the largest and smallest dimensions [16].

### Detection of Tumor-Infiltrating Lymphocytes and Cytokines

Tumor infiltrating lymphocytes (TILs) and cytokines in tumor tissues were measured by flow cytometry and immunofluorescence staining. For flow cytometry, extracted tumors were digested by incubating with 1 mg mL^−1^ collagenase (Sigma-Aldrich) in RPMI-1640 medium and the resulting cell suspensions were stained with a mixture of FITC-conjugated anti-mouse CD3 antibody and APC-conjugated anti-mouse CD8a antibody (BioLegend; catalog #100712, clone 53–6.7, lot #B266721) for 1 h. TIL function was assessed by staining cell suspensions with FITC-conjugated anti-mouse CD3 antibody, Alexa Fluor 647-conjugated anti-human/mouse granzyme B antibody (BioLegend; catalog #515406, clone GB11, lot #B233111), and PE-conjugated anti-mouse IFN-γ antibody (BioLegend; catalog #505808, clone XMG1.2, lot #B265789) for 1 h.

For immunofluorescence staining, tumors were first extracted and fixed with 4% formaldehyde for 4 h at RT. The tissue was then embedded in OCT and cryosectioned at a thickness of 10 μm using a Leica CM 3050 S microtome (Leica). After tissue permeabilization, sections were incubated with PerCP/cyanine 5.5-conjugated anti-CD8 antibody (BioLegend; catalog #100734; lot #B277115), PE-conjugated anti-IFN-γ antibody, and Alexa Fluor 647-conjugated anti-human/mouse granzyme B antibody for 12 h at 4 °C. The tissue was then washed three times with PBS, followed by staining with DAPI. Tissue fluorescence was observed using a confocal laser-scanning microscope (LSM 5 Exciter; Carl Zeiss).

### In Vivo Toxicity Study

Five-week-old C57BL/6 mice were subcutaneously injected twice with various nanoparticles. Two days after first injections, whole blood and serum samples were collected for analysis of hematological parameters regarding to white blood cell (WBC), red blood cell (RBC), hemoglobin (Hb), hematocrit (HCT), mean corpuscular volume (MCV), mean corpuscular hemoglobin (MCH), mean corpuscular hemoglobin concentration (MCHC), neutrophil, lymphocyte, monocyte, eosinophil, alanine aminotransferase (ALT), aspartate transaminase (AST), blood urea nitrogen (BUN) and creatinine. Organs (liver, lung, heart, spleen and kidney) were collected for hematoxylin and eosin staining.

### Statistics

Two-side analysis of variance (ANOVA) with post hoc Student–Newman–Keuls test was used for statistical analyses. Experimental data were analyzed with SigmaStat software (version 12.0, Systat Software). A *P*-value less than 0.05 was considered statistically significant.

## Results and Discussion

### Characterization of aCD3/F/ANs

**Fig. 2:**
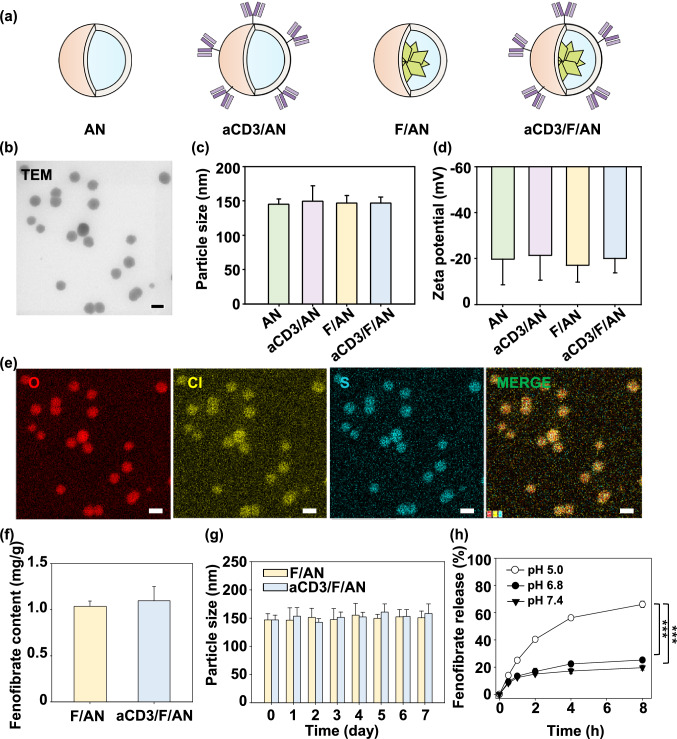
Characterization of nanoparticles. **a** Schematic illustration of ANs, aCD3/ANs, F/ANs and aCD3/F/ANs. **b** Morphology of aCD3/F/ANs, observed by TEM. Scale bar: 200 nm. **c** Mean particle size of different nanoparticles, determined using DLS. **d** Zeta potential of nanoparticles, measured by laser Doppler microelectrophoresis. **e** Characterization of aCD3/F/ANs with respect to the elements oxygen, chloride and sulfur, conducted by STEM-EDS. Scale bar: 200 nm. **f** Amount of fenofibrate encapsulated in nanoparticles, quantified by HPLC. **g** Particle sizes of F/ANs and aCD3/F/ANs in PBS, monitored for 7 days. **h** Release of fenofibrate from aCD3/F/ANs, quantified by HPLC under different pH conditions (***P < 0.001)

aCD3/F/ANs were characterized based on the physicochemical features of morphology, size, and surface charge. The structures of the various nanoparticle preparations are illustrated in Fig. 2a. Hydrophobic fenofibrate was encapsulated in self-assembled AP nanoparticles, and the surfaces of F/ANs were chemically modified with an anti-CD3 antibody, resulting in aCD3/F/ANs. TEM revealed that aCD3/F/AN particles were spherical in shape (Fig. 2b), and dynamic light scattering data showed that the sizes of aCD3/F/ANs did not significantly differ compared with those of F/ANs (Fig. 2c). The surface zeta potentials of all nanoparticles were negative regardless of antibody modification (Fig. 2d). EDS-TEM showed the presence of elemental sulfur in aCD3/F/ANs, reflecting the presence of the anti-CD3 antibody (Fig. 2e). The amount of fenofibrate per nanoparticle was not significantly different between F/ANs and aCD3/F/ANs (Fig. 2f). F/ANs and aCD3/F/ANs retained their stability in PBS for at least 7 days (Fig. 2g). The in vitro release of fenofibrate from aCD3/F/ANs was pH-dependent. At pH 7.4 and 6.8, less than 20% of fenofibrate was released from nanoparticles over 8 h. However, at pH 5.0, the release of fenofibrate from aCD3/F/ANs was greater than 60% at 8 h (Fig. 2h).

### Uptake of aCD3/F/ANs by T Cells

**Fig. 3:**
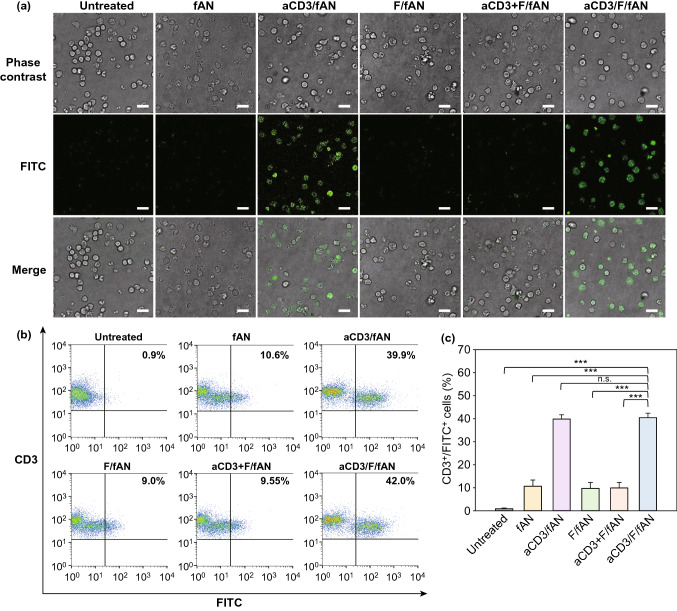
Uptake of nanoparticles by T cells. Mouse spleen-derived T cells were incubated with various fenofibrate-containing nanoparticle preparations. After 24 h, cellular uptake of nanoparticles was visualized by confocal microscopy **a** and quantified by flow cytometry **b, c** (***P < 0.001, n.s., not significant). Scale bar: 10 μm

The uptake of aCD3/F/ANs by T cells was monitored by confocal microscopy (Fig. 3a) and flow cytometry (Fig. 3b, c) using fluorescent dye-labeled nanoparticles. Confocal fluorescence microscopy revealed negligible uptake of F/fANs by T cells. Similarly, there was no notable uptake of a physical mixture of anti-CD3 antibody and fANs. In contrast, treatment with aCD3/F/fANs resulted in higher uptake of nanoparticles by T cells. Consistent with confocal microscopy images, flow cytometry showed that the fluorescence-positive T cell population was highest after treatment with aCD3/F/fANs (Fig. 3b), which resulted in a 3.8-fold higher fluorescence-positive T cell population compared with that observed in other treatment groups (Fig. 3c).

### Effect of aCD3/F/ANs on the Expression of Fatty Acid Metabolism-Related Genes and Lipid Uptake in T Cells

**Fig. 4:**
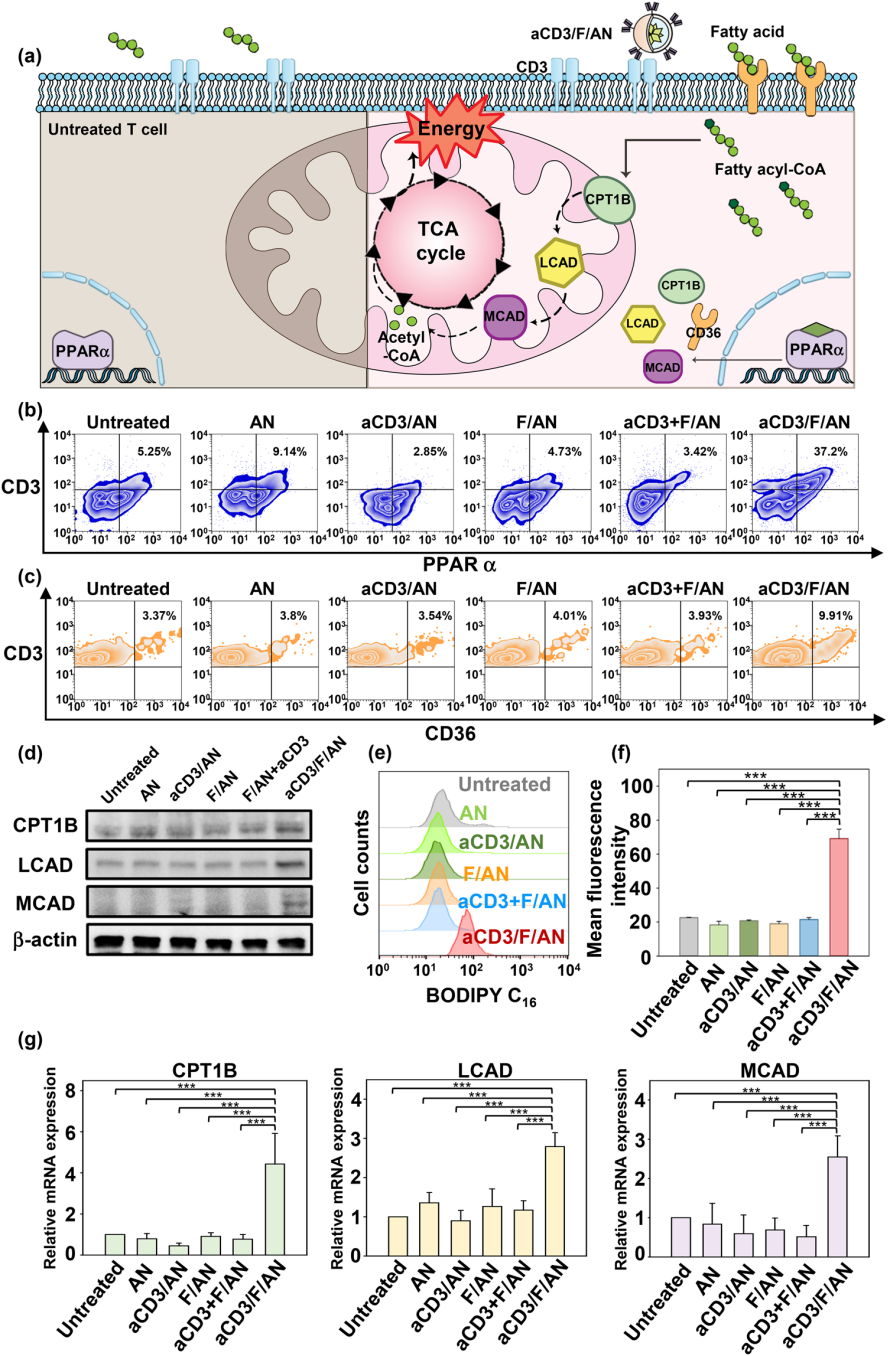
Fatty acid metabolism-associated gene expression and lipid uptake in T cells. **a** The cellular mechanism underlying aCD3/F/AN-induced enhancement of lipid metabolism is illustrated. The left half of the schematic cell depicts the untreated condition, and the right half shows treatment with aCD3/F/ANs. aCD3/F/AN treatment is proposed to activate PPARα in T cells, leading to over-expression of fatty acid metabolism-associated proteins, including CD36 (fatty acid translocase), CPT1B, LCAD and MCAD. The increased fatty acid metabolism in mitochondria generates higher energy via the TCA cycle. **b** The corresponding levels of PPARα protein in T cells (PPARα^+^/CD3^+^), measured by flow cytometry. **c** The levels of CD36 protein in T cells (CD36^+^/CD3^+^), measured by flow cytometry. **d** Protein expression levels of CPT1B, LCAD and MCAD in T cells were measured by western blot. **e, f** T cells treated with different nanoparticle preparations were incubated with fluorescent lipid BODIPY C_16_, and the association of fluorescent lipid with T cells was determined by flow cytometry (**e**) and expressed as mean fluorescence intensity (**f**) (***P < 0.001). **g** mRNA expression levels of the fatty acid metabolism-associated genes, CPT1B, LCAD and MCAD, determined by RT-PCR (***P < 0.001)

Treatment of T cells with aCD3/F/ANs affected fatty acid oxidation-related gene expression levels and lipid uptake. The cellular mechanism underlying PPARα-mediated lipid metabolism enhancement is shown in Fig. 4a. To evaluate metabolic reprogramming quantitatively, the expression levels of PPARα in T cells were measured by flow cytometry. In the T cells treated with aCD3/F/ANs, the highest expression of PPARα was observed at protein level (Fig. 4b) and mRNA level (Fig. S4b). The PPARα-positive T cell population increased by more than 7.2-fold upon treatment with aCD3/F/ANs compared with the untreated group. In addition, the expression level of a fatty acid translocase CD36, which exists on cell membrane, was also evaluated by flow cytometry. The expression of CD36 was 2.2-fold higher in the aCD3/F/AN group compared with the F/AN group (Figs. 4c and S4e). Western blot data showed that aCD3/F/ANs significantly increased the protein expression levels of CPT1B, LCAD, and MCAD compared to untreated and other groups (Figs. 4d and S5). Consistently, mRNA level of CPT1B, LCAD and MCAD was increased by 4.4-fold, 2.5-fold and 2.8-fold, respectively, compared with untreated cells (Fig. 4g). Since one consequence of the observed changes in gene expression is an increased rate of lipid uptake, we measured lipid uptake by T cells using fluorescent dye-labeled lipid, BODIPY C_16_. Compared with untreated cells, T cells treated with ANs, aCD3/ANs, F/AN or with a mixture of anti-CD3 antibody and F/ANs (aCD3 + F/ANs) showed no significant change in lipid uptake by T cells (Fig. 4e). In contrast, treatment of T cells with aCD3/F/ANs increased lipid uptake 3.1-fold compared with untreated cells (Fig. 4f).

### T Cell Mitochondrial Activation by aCD3/F/ANs

**Fig. 5:**
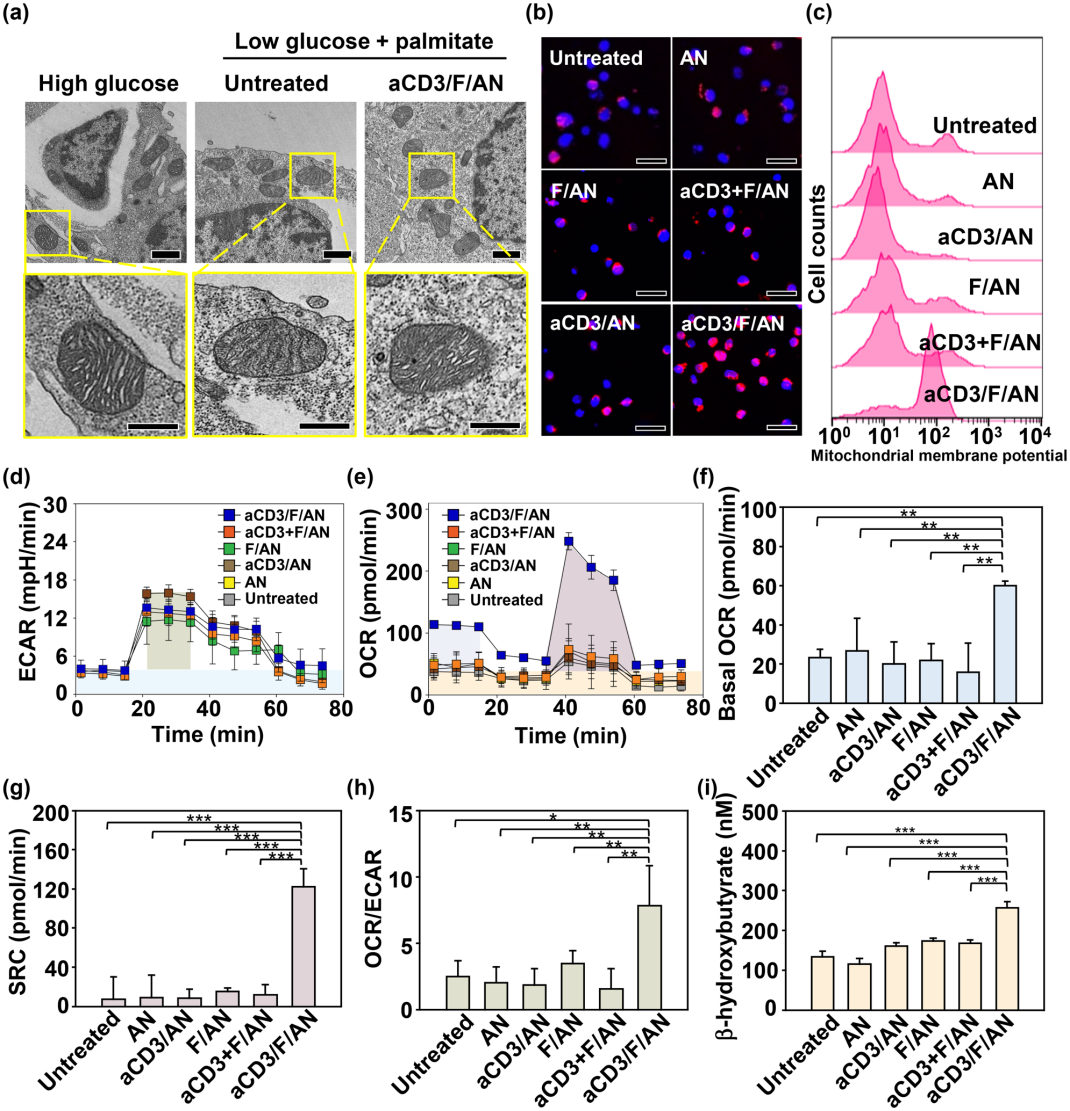
Mitochondrial morphology, membrane potential and fatty acid metabolism in T cells. T cells were treated with aCD3/F/ANs in low-glucose medium supplemented with palmitate as a lipid source. **a** TEM showing mitochondria of T cells in high-glucose medium, low-glucose medium untreated or treated with aCD3/F/AN. Scale bar: 1 μm (upper panels) and 500 nm (lower panels). **b** Mitochondrial membrane potential, assessed using MitoTracker Orange CMTMRos and visualized by confocal fluorescence microscopy. **c** Populations of T cells with increased mitochondrial membrane potentials, quantified by flow cytometry. **d, e** ECAR (**d**) and OCR (**e**), measured using a Seahorse XFp analyzer. **f–h** Basal OCR (**f)**, SRC (**g)** and OCR/ECAR **h** values, obtained based on ECAR and OCR values (*P < 0.05, **P < 0.01). **i** β-Hydroxybutyrate secretion levels (***P < 0.001)

Mitochondrial functions in T cells, including fatty acid metabolism, were modulated by treatment with aCD3/F/ANs. To mimic the glucose-deficient tumor microenvironment, we incubated T cells in low-glucose medium. In low-glucose environment containing palmitate as a lipid source, untreated T cells or T cells treated with nanoparticles such as AN, aCD3/AN and F/AN showed poorly-defined mitochondrial cristae with shrunken morphologies compared to the mitochondrial of T cells in high-glucose condition (Figs. 5a and S6). In contrast, T cells treated with aCD3/F/ANs showed mitochondrial structures with clear cristae, similar to those observed in high glucose environment (Fig. 5a).

In addition to altering the morphology of mitochondria, aCD3/F/ANs increased mitochondrial membrane potential, with fluorescent dye staining revealing the highest mitochondrial membrane potential in T cells treated with aCD3/F/ANs (Fig. 5b). Under low-glucose conditions, mitochondrial membrane potential did not differ among groups (Fig. S7a, b). However, treatment with aCD3/F/ANs in palmitate-supplemented low-glucose medium expanded the population of T cells with increased mitochondrial membrane potential by more than 6.3-fold compared with other treatment groups (Fig. 5c).

aCD3/F/AN-mediated delivery of fenofibrate to T cells enhanced lipid metabolism and mitochondrial activity. However, the ECAR value, an indicator of glycolysis, showed little difference among groups, suggesting similar levels of glycolysis in all groups (Fig. 5d). In contrast to ECAR, basal OCR, an indicator of mitochondrial respiration, was significantly higher in the aCD3/F/AN-treatment group compared with other groups (Fig. 5e, f). In addition, spare respiratory capacity (SRC), which is related to the extra ATP capacity produced by mitochondrial metabolism during a sudden increase in energy demand (calculated by subtracting minimal respiration from maximal respiration), was highest in the aCD3/F/AN-treatment group, reaching a level eightfold higher than that in the F/AN group (Fig. 5g). Moreover, the aCD3/F/AN group exhibited the highest ratio of OCR to ECAR, with a value 2.2-fold higher than that of the F/AN-treated group (Fig. 5h). Consistent with the observed enhancement in lipid metabolism, aCD3/F/AN treatment significantly increased the production of β-hydroxybutyrate, a major lipid metabolite (Fig. 5i), increasing it by 2.2-fold upon palmitate supplementation compared with F/ANs. In contrast, β-hydroxybutyrate levels were not significantly changed by AN, F/AN, aCD3/AN or aCD3 + F/AN treatment in the presence or absence of supplemental palmitate (Fig. S8a).

### In Vitro Proliferation of Metabolically Reprogrammed T Cells

**Fig. 6:**
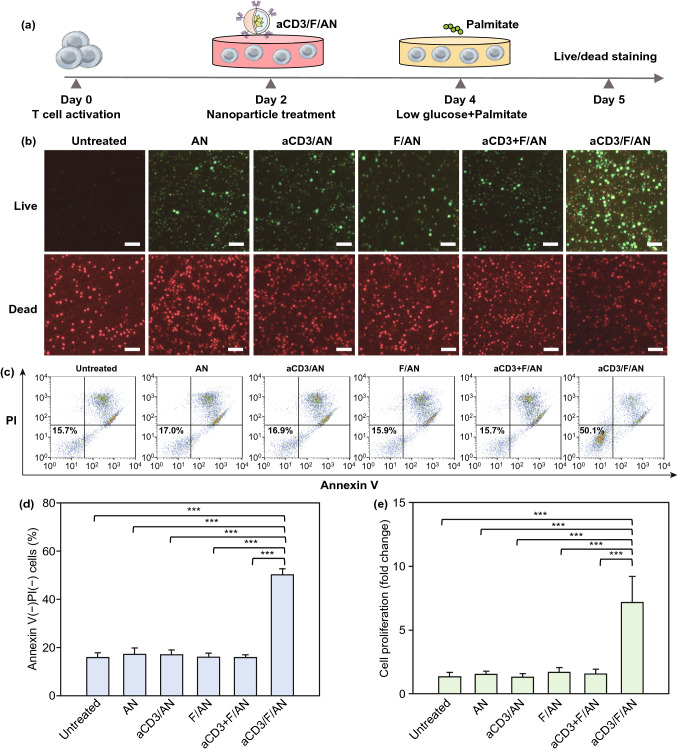
Enhanced T cell survival and proliferation induced by metabolic reprogramming. T cells were activated and treated with various nanoparticle preparations and incubated under glucose-limiting conditions, with or without a lipid source. **a** Illustration of the experimental scheme. **b** Live and dead T cells, visualized by fluorescent dye staining. Scale bar: 10 μm. **c, d** T cells were stained with annexin V and PI, and the annexin V^–^/PI^–^population was quantified for each group (***P < 0.001). **e** Proliferation of T cells, measured using WST-1 assays (***P < 0.001)

Treatment with aCD3/F/ANs affected the survival and proliferation of T cells. In these experiments, T cells were treated with various nanoparticle formulations under glucose-deficient conditions. In some experiments, low glucose medium was supplemented with palmitate as a lipid source. Fluorescent dye-based live/dead assays revealed that, in such a glucose-deficient, but palmitate-replete, environment, the aCD3/F/AN-treatment group showed the highest fraction of live T cells (Fig. 6b) and a significantly higher annexin V^–^/PI^–^ cell population compared with other treatment groups (Fig. 6c, d). In addition, upon palmitate supplementation, the proliferation of aCD3/F/AN-treated T cells was 5.4-fold higher than that in the untreated group (Fig. 6e).

### In Vitro Cancer Cell-Killing Activity of aCD3/F/AN-Treated T Cells

**Fig. 7:**
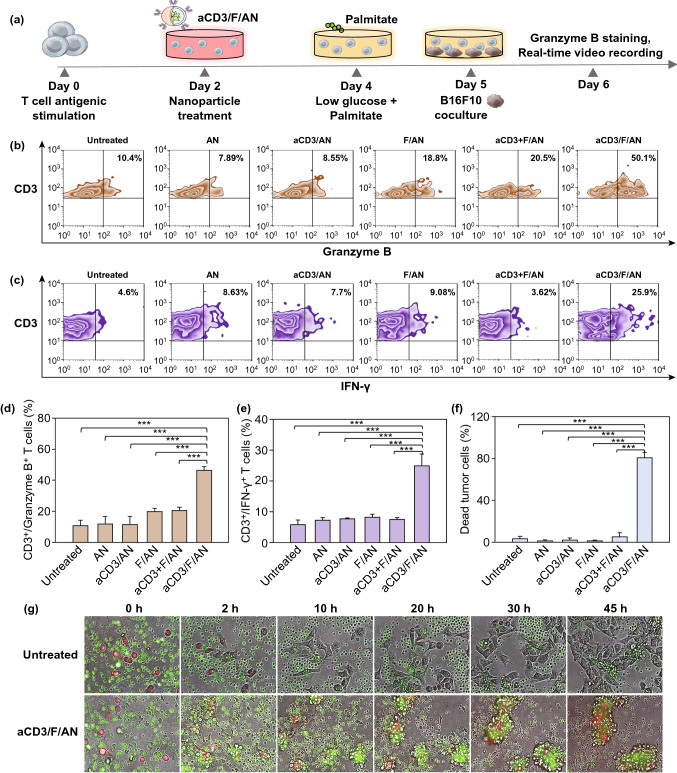
In vitro anticancer activity of aCD3/F/AN-treated T cells. T cells were treated with various nanoparticle preparations and co-incubated with B16F10 cells under glucose-limiting conditions in the presence of palmitate as a lipid source. **a** Illustration of the experimental scheme. **b, c** Flow cytometry data of T cells positive with granzyme B (**b**) and IFN-γ (**c**). **d, e** The populations of T cells positive with granzyme B (**d**) and IFN-γ (**e**) were quantified. (***P < 0.001). **f** Dead cancer cell population, quantified by flow cytometry (***P < 0.001). **g** Lysis of cancer cells by T cells, recorded in real time. Cancer cells were labeled with red fluorescent dye, whereas T cells were labeled with green fluorescent dye. Real-time video recordings of untreated T cells or T cells treated with aCD3/F/ANs are shown in Supplementary video S1 and S2, respectively

The treatment with aCD3/F/ANs enhanced the cancer cell-killing activity of T cells. Upon co-incubation of T cells with B16F10 melanoma cells, the levels of granzyme B and IFN-γ in aCD3/F/AN-treated T cells were 2.3- and 3.0-fold higher compared with those in the F/AN-treated group, respectively (Fig. 7b–e). The cancer cell-killing activity of T cells treated with ANs, F/ANs, aCD3/AN or aCD3 + F/AN was not different from those of untreated T cells (Fig. 7f). However, the population of dead cancer cells following incubation with aCD3/F/AN-treated T cells was 15.6-fold higher than that following incubation with T cells treated with aCD3 + F/ANs (Figs. 7f and S9). Real time video recordings further showed that untreated T cells did not attack B16F10 cells (Supplementary Video S1), whereas aCD3/F/AN-treated T cells vigorously attacked B16F10 cells (Supplementary Video S2). Still images of untreated T cells and aCD3/F/AN-treated T cells captured at various time points are shown in Fig. 7g.

### In Vivo Distribution of aCD3/F/ANs to T Cells in Tumor Tissues

**Fig. 8:**
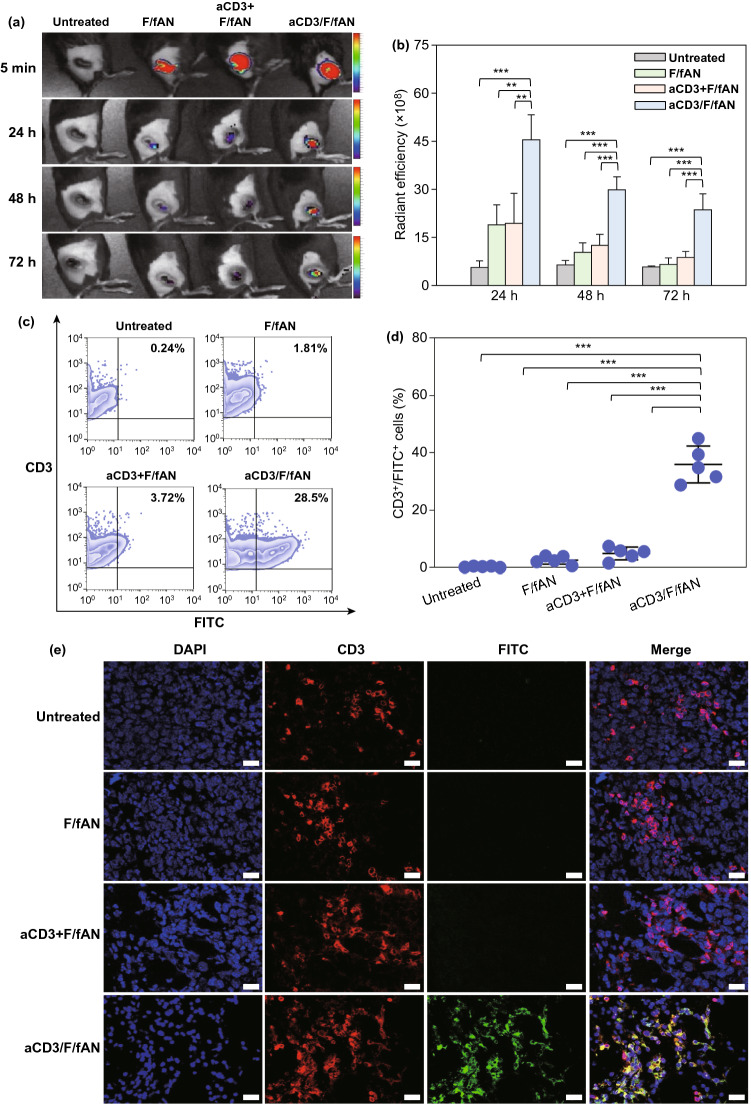
In vivo T cell-targeting ability of aCD3/F/ANs. T cell targeting ability was evaluated by intratumorally injecting mice (n = 3) with various nanoparticle preparations containing fenofibrate. **a** In vivo fluorescence at tumor sites, visualized at different time points. **b** Quantification of fluorescence in tumor sites at different time points (**P < 0.01, ***P < 0.001). **c** Uptake of nanoparticles in tumor-resident T cells, analyzed by flow cytometry. **d** CD3^+^FITC^+^ T cell population, quantified for each treatment group (***P < 0.001). **e** Colocalization of nanoparticles (green) with CD3^+^ T cells (red), visualized by confocal microscopy. Scale bars: 20 μm

The in vivo distribution of nanoparticles to T cells in tumor tissues was visualized by determining colocalization with T cells in fluorescence images. aCD3/F/fANs showed the highest retention in tumor tissues compared with F/fANs and the aCD3 + F/AN group (Fig. 8a). An intensity analysis showed that the distribution of nanoparticles to tumor tissues over 72 h post-dose was highest in the group treated with aCD3/F/fANs (Fig. 8b). At 72 h, ex vivo fluorescence imaging of extracted tumor tissues revealed that aCD3/F/fANs were retained to a higher extent than other nanoparticle preparations (Fig. S9). Uptake of nanoparticles by T cells in tumor tissues was tested by assessing colocalization of nanoparticles and the FITC^+^CD3^+^ T cell population. The colocalization of nanoparticles to CD3^+^ T cells was highest in the aCD3/F/fAN-treatment group, which exhibited 7.6-fold greater colocalization than the F/fAN group at 24 h (Fig. 8c, d). Tumor tissue staining showed higher colocalization of aCD3/F/fANs with CD3^+^ T cells in tumor tissues (Fig. 8e).

### Reprogramming of In Vivo Fatty Acid Metabolism in aCD3/F/AN-Treated T Cells

**Fig. 9:**
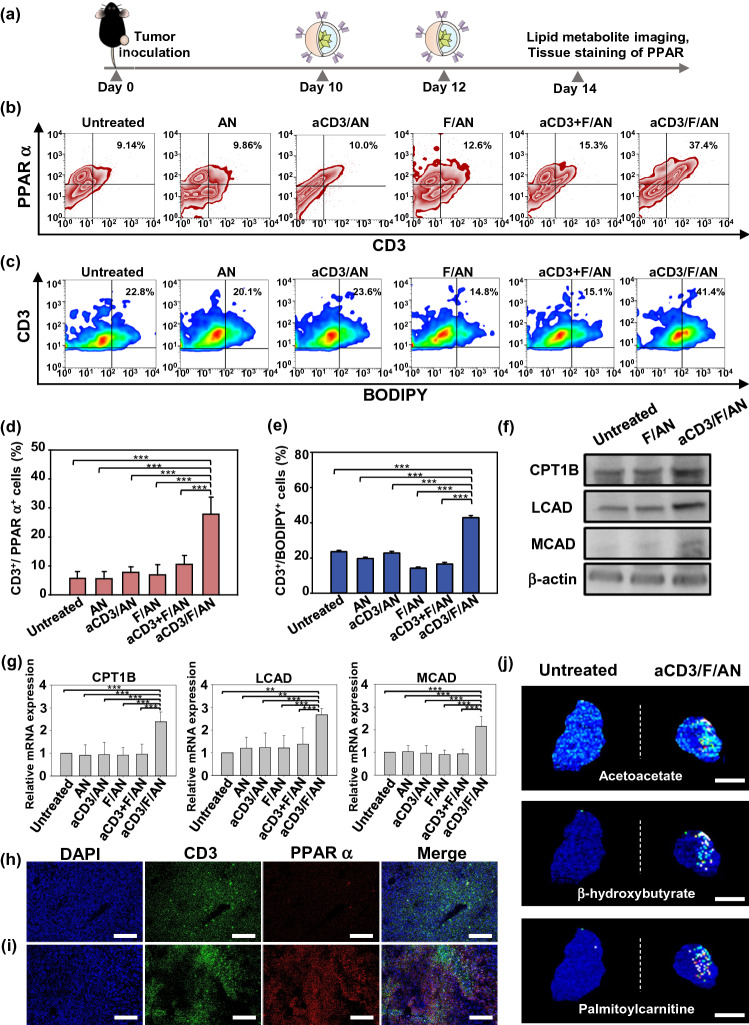
In vivo PPARα expression and lipid uptake by T cells in tumor tissues. **a** B16F10 tumor-bearing mice were intratumorally injected with various nanoparticle preparations on days 10 and 12 after tumor inoculation. On day 13, BODIPY C_16_ was intratumorally injected, and on day 14, tumor tissues were extracted and analyzed. **b**–**e** PPARα expression level in T cells **(b, d)** and BODIPY C_16_-positive CD3 T cells **(c, e)**, analyzed by flow cytometry (***P < 0.001). **f** Protein expression levels of CPT1B, LCAD and MCAD in tumor infiltrating T cells were determined by western blot. **g** mRNA expression levels of the fatty acid metabolism-associated genes CPT1B, LCAD and MCAD in tumor infiltrating T cells were determined by RT-PCR (***P < 0.001). **h, i** Confocal microscopic images of untreated (**h**) and aCD3/F/AN-treated tumor tissue (**i**) were immunostained with anti-CD3 (green) and anti-PPAR α (red) antibody and counterstained with DAPI. Scale bars: 100 μm. **j** Tumor tissue distribution of acetoacetate (m/z = 103.09), β-hydroxybutyrate (m/z = 105.10) and palmitoylcarnitine (m/z = 400.60), as visualized by MALDI imaging. Scale bar: 5 μm

The intratumoral administration of aCD3/F/ANs modulated in vivo PPARα expression, lipid uptake and metabolism by T cells. The in vivo experimental scheme is illustrated in Fig. 9a. Flow cytometry showed that PPARα-positive (Fig. 9b) and BODIPY C_16_-positive (Fig. 9c). T cell populations were increased to a greater extent in the aCD3/F/AN-treatment group. Expression of PPARα was increased 4.1-fold and 2.6-fold in this group compared with F/AN- and aCD3 + F/AN-treatment groups, respectively (Fig. 9d). Uptake of BODIPY C_16_ lipid was almost twofold higher in the aCD3/F/AN-treatment group than in other groups (Fig. 9e). Mice treated with aCD3/F/AN showed significantly increased protein expression and mRNA levels of CPT1B, LCAD and MCAD compared with untreated group (Fig. 9f, g). Immunostaining revealed distinct PPARα expression in CD3^+^ T cells in tumor tissues, as demonstrated by colocalization of CD3 and PPARα staining (Fig. 9h, i). MALDI imaging revealed enhanced levels of the fatty acid oxidation metabolites, acetoacetate, β-hydroxybutyrate and palmitoylcarnitine, in T cells treated with aCD3/F/ANs (Fig. 9j).

### In Vivo Antitumor Efficacy of Metabolically Reprogrammed T Cells

**Fig. 10:**
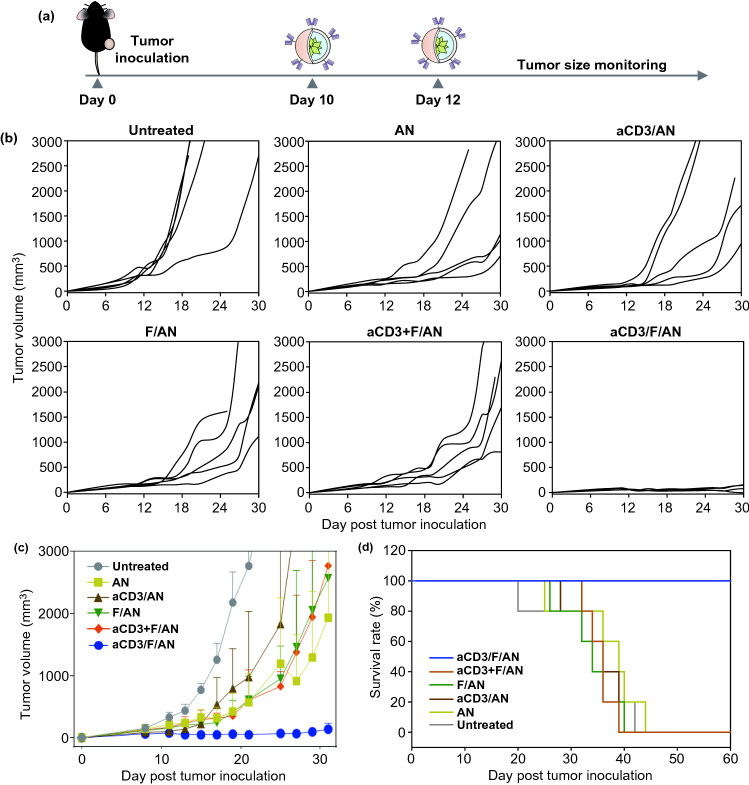
Antitumor effects of aCD3/F/ANs in vivo. **a** B16F10 tumor-bearing mice were intratumorally injected with various nanoparticle preparations on days 10 and 12 after tumor inoculation. **b, c** Antitumor efficacy, determined by measuring tumor volume. **d** Survival rate in each mouse group, monitored for 60 days after tumor inoculation

To assess the effects of aCD3/F/ANs on tumor growth and the survival of mice, we intratumorally injected B16F10 tumor-bearing mice with various nanoparticle preparations twice, once on day 10 after tumor inoculation and again on day 12 (Fig. 10a). aCD3/F/AN treatment exerted significantly higher tumor growth-inhibitory effects than treatment with other nanoparticle preparations (Fig. 10b, c). Notably, the survival rate of mice treated with aCD3/F/ANs was 100% up to day 60. In contrast, no mice survived beyond 44 days after tumor inoculation in any other treatment group (Fig. 10d).

**Fig. 11:**
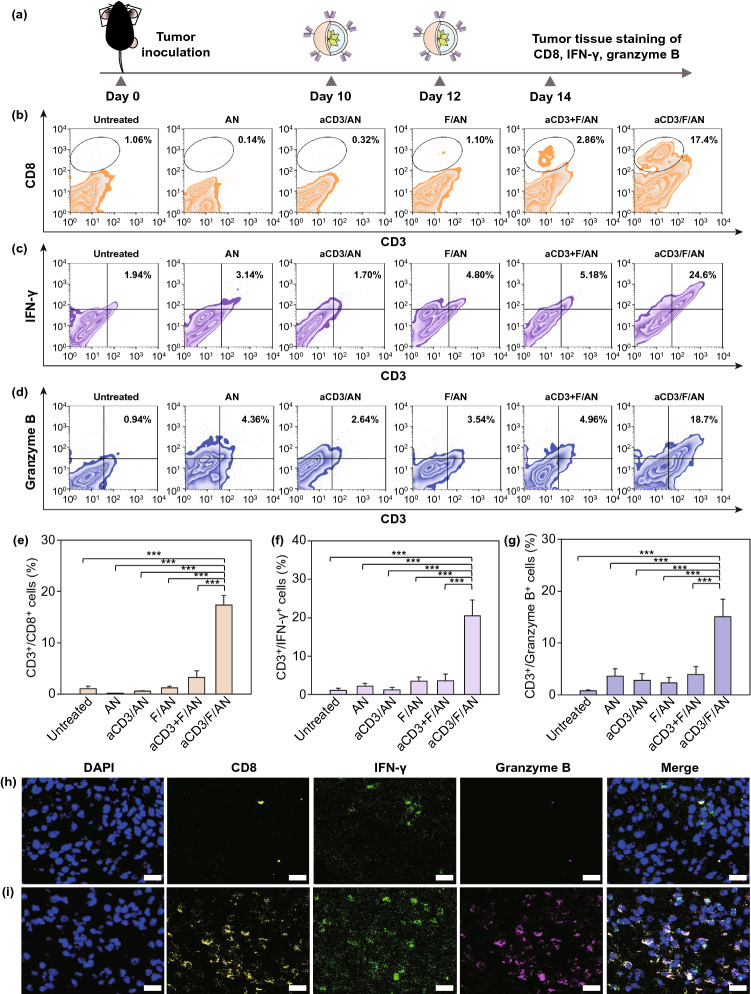
In vivo cytokine production by T cells in tumor tissues. **a** B16F10 tumor-bearing mice were injected with nanoparticle preparations on days 10 and 12 after tumor inoculation. On day 14, tumor tissues were extracted for analysis. **b-d** Infiltrating CD8^+^ T cells (**b**), IFN-γ–expressing T cells (**c**) and granzyme B–expressing T cells (**d**) among tumor tissue CD3^+^ T cells, determined by flow cytometry (***P < 0.001). Populations of CD3^+^/CD8^+^ cells (**e)**, CD3^+^/ IFN-γ^+^ cells (**f)** and CD3^+^/granzyme^+^ cells (**g)** are shown. **h, i** In untreated (**h**), and aCD3/F/AN-treated mice (**i**), tumor tissues were extracted and immunostained with anti-CD8 (yellow), anti-IFN-γ (green) and anti-granzyme B (light purple) antibodies and counterstained with DAPI. Scale bar: 20 μm

To test whether the observed antitumor efficacy was attributable to the enhanced function of metabolically reprogrammed T cells, we quantified tumor-infiltrating CD8^+^ T cells (Fig. 11b). The CD8^+^ T cell population in tumor tissues was significantly increased in mice treated with aCD3/F/ANs, which exhibited a CD8^+^ T cell population in tumor tissues 14.8-times higher than that in F/AN-treated mice (Fig. 11b, e). In vivo treatment with aCD3/F/ANs affected expression levels of IFN-γ and granzyme B in T cells. Specifically, IFN-γ–expressing T cells accounted for 20.5% ± 4.1% of tumor T cells in the aCD3/F/AN-treated group—a more than fivefold increase compared with F/AN- and aCD3 + F/AN-treatment groups (Fig. 11c, f). Similar to the IFN-γ expression pattern, the highest percentage of granzyme B–expressing T cells was observed for the group treated with aCD3/F/ANs, which showed more than a 3.9-fold increase in levels compared with other treatment groups (Fig. 11d, g). An immunohistochemical analysis of tumor tissues showed no notable infiltration of CD8^+^ T cells in tumor tissues in the untreated mice (Fig. 11h). However, considerable infiltration of CD8^+^ T cells in tumor tissues and secretion of IFN-γ and granzyme B were observed in tumors treated with aCD3/F/ANs (Fig. 11i).

Here, we demonstrated that enhanced delivery of fenofibrate to T cells modulated the fatty acid metabolism of T cells and stimulated their cancer cell-killing activity. T cell-directed delivery of fenofibrate was achieved by modification of the surfaces of fenofibrate-loaded nanoparticles with an anti-CD3 antibody. Uptake of aCD3/F/ANs by T cells increased mitochondrial function and the expression of fatty acid metabolism-related genes, including PPARα. T cells metabolically reprogramed by aCD3/F/ANs efficiently killed B16F10 melanoma cells in vitro, and an in vivo study showed that aCD3/F/AN treatment prevented the growth of tumors and increased the production of various cytokines.

In this study, we entrapped fenofibrate in ANs and modified the surfaces of F/ANs with anti-CD3e f(ab’)2 antibody fragments. The nanoparticle is based on the amphiphilic derivative of poly (γ-glutamic acid) which is natural biopolymer produced from Bacillus species [30]. Due to the biocompatibility and biodegradability, poly (γ-glutamic acid)-based biomaterials have been studied for delivery systems of various active substances. Poly (γ-glutamic acid) and gold nanocluster hybrid nanoparticles were reported for receptor-mediated cancer cell delivery and photothermal therapy [31]. Poly (γ-glutamic acid) coating of doxorubicin-loaded mesoporous silica nanoparticle was shown to increase the delivery to cancer cell and enhance the anticancer effect of doxorubicin [32]. Amphiphilic phenylalanine derivative of poly (γ-glutamic acid) was found to self-assemble polymeric micelles and entrap hydrophobic anticancer drugs in the hydrophobic phenylalanine core parts [22]. Moreover, the carboxylic groups at the surfaces of amphiphilic poly (γ-glutamic acid)-based nanoparticles enable the surface modification with other functional molecules such as targeting ligand chemicals and antibodies [33].

Here, fenofibrate was encapsulated inside of the AN matrix, which is thought to provide a hydrophobic core by virtue of the phenylalanine moieties of AP. The release of fenofibrate from aCD3/F/ANs at lower pH values might be attributable to protonation of carboxyl groups in AP. The CD3 receptor, which has been used as a marker of T cells [34], was chosen as a target because it is overexpressed on T cells and has been reported to induce receptor-mediated endocytosis upon binding of nanoparticles [33]. Indeed, we observed enhanced uptake of aCD3/F/ANs by T cells compared with plain F/ANs both in vitro (Fig. 3) and in vivo in B16F10 tumor tissues (Fig. 8e). The comparable T cell uptake of aCD3/ANs (Fig. 3) supports the major role of CD3 antibody fragments in T cell delivery.

The CD3 antibody-mediated delivery enabled aCD3/F/ANs to show high selectivity to T cells without side effect to other cells. Our data support that the expression levels of CD3 were notably higher in T cells compared to other cells (Fig. S1). In line with the CD3 expression levels, the uptake of aCD3/F/ANs was significantly higher in T cells compared to other cells such as BMDC, BMDM, MLg, MRC-5, and 293 T (Fig. S2). Although the cellular uptake patterns were affected by the expression levels of CD3, the viability of cells were not affected by the types of cells, and nanoparticles. In all cells tested, the viability was retained after treatment with various nanoparticles (Fig. S3).

Consistently, our in vitro and in vivo toxicity study demonstrated that aCD3/F/AN did not induce any cytotoxicity (Fig. S3) or systemic side effects (Fig. S12). In vivo safety study revealed that the repeated injection of mice with aCD3/F/ANs did not alter histology (Fig. S12a) or organ functions, showing similar levels of ALT, AST, BUN and creatinine compared to untreated group (Fig. S12b). Moreover, the levels of ALT, AST, BUN in the group treated with aCD3/F/ANs did not significantly differ from those of untreated group.

CD3 receptor-mediated enhanced delivery of fenofibrate to T cells was shown to induce the expression of fatty acid uptake- and metabolism-related genes (Fig. 4). We found that uptake of fluorescent lipid by T cells was increased only in aCD3/F/AN-treated T cells, and not T cell treated with other nanoparticle preparations (Fig. 4f, g). The higher uptake of fluorescent lipids by aCD3/F/AN-treated T cells may be attributable to upregulation of fatty acid transport, consistent with a previous report that PPARα agonists induce up-regulation of the fatty acid transporter, CD36 [35]. Indeed, we observed that expression of CD36 was highest in T cells treated with aCD3/F/ANs (Fig. 4c). CD36, known as fatty acid translocase, functions in mediating the entry of extracellular fatty acids into the cytosol of cells [36].

Fatty acids entering T cells via CD36 can be transported to mitochondria, where they feed into the TCA cycle. We observed that treatment with aCD3/F/ANs increased the expression of CPT1B, LCAD, and MCAD (Fig. 4d, e). CPT1B is known to increase the transport of fatty acids from the cytosol to mitochondria [37], whereas LCAD and MCAD are involved in the β-oxidation of fatty acids [38, 39]. The increased expression of fatty acid oxidation-related genes is attributable to the ability of the pharmacological agent fenofibrate to activate PPARα [40]. PPARα, the molecular target of fenofibrate, is a key transcriptional regulator of fatty acid oxidation-related genes. In association with enhanced expression of PPARα, we observed increased expression of the downstream fatty acid oxidation-related genes CPT1B, LCAD and MCAD. Consistent with this, it has been reported that expression of CPT1B, LCAD and MCAD is induced by activation of the transcription factor, PPARα [25].

Our observations support the interpretation that uptake of aCD3/F/ANs by T cells activates fatty acid metabolism, but not glycolysis (Fig. 5). The significantly higher OCR and ECAR ratio in aCD3/F/AN-treated T cells further supports the conclusion that fenofibrate affected fatty acid metabolism. T cells were metabolically reprogrammed to take up and use fatty acids as an energy source. In the presence of a fatty acid energy source, the reprogrammed T cells maintained a higher metabolic state and enhanced mitochondrial function, as evidenced by the levels of lipid metabolites in aCD3/F/AN-treated T cells. Because our data showed that treatment with aCD3/ANs alone did not affect fatty acid metabolism, it is unlikely that fatty acid metabolism is affected by nanoparticles per se; instead enhanced fatty acid metabolism is attributable to activation of PPARα by fenofibrate. Consistent with this, it has been reported that fenofibrate activates PPARα in various cell types, including endothelial cells [41], cardiomyocytes [42], and adipocytes [43].

The enhanced mitochondrial functions of aCD3/F/AN-treated T cells may be attributable to the prolonged survival, proliferation and effector functions of T cells (Figs. 6 and 7). T cells are known to undergo metabolic stress and exhibit mitochondrial dysfunction in glucose-deficient tumor microenvironments [44]. Our TEM imaging revealed a shrunken mitochondrial morphology and decreased mitochondrial membrane potentials in glucose-deprived T cells (Fig. 5). Mitochondrial membrane potential is known to play a crucial role in ATP production and maintenance of cellular functions [45]. Treatment with aCD3/F/ANs restored clear cristae structures and membrane potentials of mitochondria. Notably, treatment with aCD3/F/ANs enhanced the cancer cell-killing effector function of T cells, likely owing to the reprogramming of T cells to use fatty acids as an alternative energy source.

Fatty acid metabolism via the TCA cycle, which can provide greater energy to T cells, may lead to the production of various cytokines, as underscored by several reports on the relationship between robust mitochondrial function and energy production in T cells on the one hand and cytokine secretion on the other. Consistent with this, we found that metabolic reprogramming of T cells by aCD3/F/ANs resulted in increased secretion of the cytokine, granzyme B (Fig. 7). It was previously reported that fenofibrate induces mitochondrial fatty acid oxidation-mediated augmentation of the effector function of T cells by enhancing granzyme B and IFN-γ secretion [21]. Other studies have also reported that mitochondrial metabolism is essential for the ability of T cells to adapt by rapidly modifying their energy requirements. It has been reported that memory CD8^+^ T cells maintain a higher mitochondrial metabolic rate through fatty acid oxidation and actively produce the cytokines, IFN-γ and IL-2 [46]; their mitochondrial function and secretion of IL-2 and TNF-α are also enhanced by acute infection [47]. Our observations are in agreement with previous findings that mitochondrial function plays a crucial role in the production of cancer-killing cytokines by T cells.

Although we targeted CD3, which is expressed in various types of T cells, our focus was mainly on the effect of metabolic manipulation on effector CD8^+^ T cells. Mitochondrial energy production is known to be critical for the ability of CD8^+^ T cells to exert their cytotoxic function. In the case of regulatory T cells, metabolic adaptations allow these cells to maintain their inhibitory immune functions with the help of FoxP3. Several studies have reported that FoxP3, acting as a master transcriptional regulator, reduces the dependence of regulatory T cells on glycolysis to favor the mitochondrial oxidation pathway, providing these cells with a metabolic advantage over CD8^+^ T cells in low-glucose environments [48, 49]. It is unlikely that the improved effector function of T cells was dependent on the alterations of FoxP3 expression. In this study, we observed that aCD3/F/AN did not significantly affect the expression of FoxP3 in T cells (Fig. S10).

The metabolic reprogramming of tumor-infiltrating T cells, as revealed by their elevated expression of PPARα in aCD3/F/AN-treated tumor tissues (Fig. 9), contributes to the anticancer efficacy of aCD3/F/ANs. An additional contributing factor is the enhanced in vivo delivery of aCD3/F/ANs to T cells in tumor tissues, which exceeded that of all other nanoparticle preparations (Fig. 8). In this study, to enhance infiltration of T cells into tumor tissues, we pretreated mice with DOX, which is reported to act as an immunogenic cell inducer [27], and MPL, used as an immunoadjuvant [28]. The enhanced infiltration and activation of T cells by aCD3/F/AN-induced metabolic reprogramming, in turn, enhanced the killing of tumor cells. This is supported by the production of the cytokines, IFN-γ and granzyme B (Fig. 11), the latter of which has been shown to be a marker of activated cytotoxic T cells [50].

In this study, we achieved the selective activation of PPARα by delivering fenofibrate only to T cells using an aCD3 antibody fragment. The selective delivery of fenofibrate can increase the amount of fenofibrate available to T cells and reduce the nonspecific distribution of fenofibrate to other tissues of the body. The importance of selective T cell targeting is supported by our demonstration that the physical combination of aCD3/ANs + F/ANs produced no meaningful alterations in metabolism in virtually all experimental settings. Importantly, the lack of significant anticancer effect of aCD3/ANs + F/ANs (Figs. 7 and 10) and the observation that aCD3/F/AN treatment completely abrogated tumor growth in B16F10 tumor-bearing mice (Fig. 10) support the importance of T cell targeted delivery of nanoparticles.

Although we targeted tumor-infiltrating T cells by aCD3/F/AN in this study, the concept of lipid metabolic reprogramming can be broadly applied to adoptive T cell transfer. Currently, the anticancer effects of chimeric antigen receptor-engineered T cells against solid tumors are limited, partly because of their diminished viability in the hypoglycemic tumor microenvironment [51]. For adoptive T cell transfer, ex vivo pretreatment of T cells with aCD3/F/ANs might be used to fortify the survival and effector functions of these cells in the tumor microenvironment.

## Conclusions

In conclusion, we have provided evidence that metabolic reprogramming of T cells can be used as a new mode of anticancer immunometabolic therapy. The enhanced T cell delivery achieved using CD3/F/ANs was shown to activate fatty acid oxidation metabolic pathways and restore mitochondrial functions of T cells in a glucose-deficient environment. Treatment with aCD3/F/ANs also increased effector functions of T cells against tumor cells. We used T cells for metabolic reprogramming in the current study, but the concept of metabolic reprogramming as a strategy for activating anticancer effector functions can be broadly applied to the design of other immunometabolic therapies against solid tumors.

## Electronic Supplementary Material

Below is the link to the electronic supplementary material.

Supplementary material 1 (PDF 1854 kb)

Supplementary material 2 (mp4 9670 kb)

Supplementary material 3 (mp4 9476 kb)

## References

[1] Li Y, Lin J, Wang P, Luo Q, Zhu F (2020). Tumor microenvironment cascade-responsive nanodrug with self-targeting activation and ROS regeneration for synergistic oxidation-chemotherapy. Nano-Micro Lett..

[2] Irvine DJ, Dane EL (2020). Enhancing cancer immunotherapy with nanomedicine. Nat. Rev. Immunol..

[3] Nam J, Son S, Park K, Zou W, Shea LD (2019). Cancer nanomedicine for combination cancer immunotherapy. Nat. Rev. Mater..

[4] Miao Y, Qiu Y, Zhang M, Yan K, Zhang P (2020). Aqueous self-assembly of block copolymers to form manganese oxide-based polymeric vesicles for tumor microenvironment-activated drug delivery. Nano-Micro Lett..

[5] Anchordoquy TJ, Barenholz Y, Boraschi D, Chorny M, Decuzzi P (2017). Mechanisms and barriers in cancer nanomedicine: addressing challenges, looking for solutions. ACS Nano.

[6] Rosenblum D, Joshi N, Tao W, Karp JM, Peer D (2018). Progress and challenges towards targeted delivery of cancer therapeutics. Nat. Commun..

[7] Meacham CE, Morrison SJ (2013). Tumour heterogeneity and cancer cell plasticity. Nature.

[8] Fernandez M, Javaid F, Chudasama V (2017). Advances in targeting the folate receptor in the treatment/imaging of cancers. Chem. Sci..

[9] Li M, Li M, Yang Y, Liu Y, Xie H (2020). Remodeling tumor immune microenvironment via targeted blockade of PI3K-γ and CSF-1/CSF-1R pathways in tumor associated macrophages for pancreatic cancer therapy. J. Control. Release.

[10] Zhang N, Liu S, Shi S, Chen Y, Xu F (2020). Solubilization and delivery of Ursolic-acid for modulating tumor microenvironment and regulatory T cell activities in cancer immunotherapy. J. Control. Release.

[11] Trinh A, Polyak K (2019). Tumor neoantigens: when too much of a good thing is bad. Cancer Cell.

[12] Brennen WN, Isaacs JT, Denmeade SR (2012). Rationale behind targeting fibroblast activation protein–expressing carcinoma-associated fibroblasts as a novel chemotherapeutic strategy. Mol. Cancer Ther..

[13] Wculek SK, Cueto FJ, Mujal AM, Melero I, Krummel MF (2020). Dendritic cells in cancer immunology and immunotherapy. Nat. Rev. Immunol..

[14] Wu TD, Madireddi S, de Almeida PE, Banchereau R, Chen YJJ (2020). Peripheral T cell expansion predicts tumour infiltration and clinical response. Nature.

[15] Kim M, Shon Y, Kim J, Oh Y (2016). Selective activation of anticancer chemotherapy by cancer-associated fibroblasts in the tumor microenvironment. J. Natl. Cancer Inst..

[16] Le Q, Suh J, Choi J, Park G, Lee J (2019). In situ nanoadjuvant-assembled tumor vaccine for preventing long-term recurrence. ACS Nano.

[17] Mu W, Chu Q, Liu Y, Zhang N (2020). A review on nano-based drug delivery system for cancer chemoimmunotherapy. Nano-Micro Lett..

[18] Binnewies M, Roberts EW, Kersten K, Chan V, Fearon DF (2018). Understanding the tumor immune microenvironment (TIME) for effective therapy. Nat. Med..

[19] Thommen DS, Schumacher TN (2018). T cell dysfunction in cancer. Cancer Cell.

[20] Li H, Bullock K, Gurjao C, Braun D, Shukla SA (2019). Metabolomic adaptations and correlates of survival to immune checkpoint blockade. Nat. Commun..

[21] Zhang Y, Kurupati R, Liu L, Zhou X, Zhang G (2017). Enhancing CD8+ T cell fatty acid catabolism within a metabolically challenging tumor microenvironment increases the efficacy of melanoma immunotherapy. Cancer Cell.

[22] Kim D, Le Q, Kim Y, Oh Y (2019). Safety and photochemotherapeutic application of poly(γ-glutamicacid)-based biopolymeric nanoparticle. Acta Pharm. Sin. B.

[23] Rodell CB, Arlauckas SP, Cuccarese MF, Garris CS, Li R (2018). TLR7/8-agonist-loaded nanoparticles promote the polarization of tumour-associated macrophages to enhance cancer immunotherapy. Nat. Biomed. Eng..

[24] Song J, Ardakani SS, So T, Croft M (2007). The kinases aurora B and mTOR regulate the G1–S cell cycle progression of T lymphocytes. Nat. Immunol..

[25] Chowdhury PS, Chamoto K, Kumar A, Honjo T (2018). PPAR-induced fatty acid oxidation in T cells increases the number of tumor-reactive CD8^+^ T Cells and facilitates anti–PD-1 therapy. Cancer Immunol. Res..

[26] Livesey SA, Linner JG (1987). Cryofixation taking on a new look. Nature.

[27] Ma Y, Adjemian S, Mattarollo SR, Yamazaki T, Aymeric L (2013). Anticancer chemotherapy-induced intratumoral recruitment and differentiation of antigen-presenting cells. Immunity.

[28] Bonam SR, Partidos CD, Halmuthur SKM, Muller S (2017). An overview of novel adjuvants designed for improving vaccine efficacy. Trends Pharmacol. Sci..

[29] Pouliot K, Corbett RB, Roix RM, Paquette SM, West K (2014). Contribution of TLR4 and MyD88 for adjuvant monophosphoryl lipid A (MPLA) activity in a DNA prime–protein boost HIV-1 vaccine. Vaccine.

[30] Ajayeoba TA, Dula S, Ijabadeniyi OA (2019). Properties of poly-γ-glutamic acid producing-bacillus species isolated from Ogi Liquor and Lemon-Ogi Liquor. Front Microbiol..

[31] Ko S, Park J, Lee Y, Lee D, Macgregor RB (2020). Biochemical reprogramming of tumors for active modulation of receptor-mediated nanomaterial delivery. Biomaterials.

[32] Du X, Xiong L, Dai S, Qiao S (2015). γ-PGA-coated mesoporous silica nanoparticles with covalently attached prodrugs for enhanced cellular uptake and intracellular GSH-responsive release. Adv. Healthc. Mater..

[33] Smith TT, Stephan SB, Moffettl HF, McKnight LE, Ji W (2017). In situ programming of leukaemia-specific T cells using synthetic DNA nanocarriers. Nat. Nanotechnol..

[34] Dong D, Zheng L, Lin J, Zhang B, Zhu Y (2019). Structural basis of assembly of the human T cell receptor–CD3 complex. Nature.

[35] Pawlak M, Lefebvre P, Staels B (2015). Molecular mechanism of PPARa action and its impact on lipid metabolism, inflammation and fibrosis in non-alcoholic fatty liver disease. J. Hepatol..

[36] Pascual G, Avgustinova A, Mejetta S, Martín M, Castellanos A (2017). Targeting metastasis-initiating cells through the fatty acid receptor CD36. Nature.

[37] L’Hortet A, Takeishi K, Lepe J, Morita K, Achreja A (2019). Generation of human fatty livers using custom-engineered induced pluripotent stem cells with modifiable SIRT1 metabolism. Cell Metab..

[38] Wang T, Cao Y, Zheng Q, Tu J, Zhou W (2019). SENP1-Sirt3 signaling controls mitochondrial protein acetylation and metabolism. Mol. Cell.

[39] Niu Z, Shi Q, Zhang W, Shu Y, Yang N (2017). Caspase-1 cleaves PPARγ for potentiating the pro-tumor action of TAMs. Nat. Commun..

[40] Lee H, Gao X, Barrasa MI, Li H, Elmes RR (2015). PPAR-a and glucocorticoid receptor synergize to promote erythroid progenitor self-renewal. Nature.

[41] Xu N, Wang Q, Jiang S, Wang Q, Hu W (2019). Fenofibrate improves vascular endothelial function and contractility in diabetic mice. Redox Biol..

[42] Nakamura M, Liu T, Husain S, Zhai P, Warren JS (2019). Glycogen synthase kinase-3a promotes fatty acid uptake and lipotoxic cardiomyopathy. Cell Metab..

[43] Shen Y, Su Y, Silva FJ, Weller AH, Colon JS (2020). Shared PPARα/γ target genes regulate brown adipocyte thermogenic function. Cell Rep..

[44] Siska PJ, Beckermann KE, Mason FM, Andrejeva G, Greenplate AR (2017). Mitochondrial dysregulation and glycolytic insufficiency functionally impair CD8 T cells infiltrating human renal cell carcinoma. JCI Insight..

[45] Sprenger HG, Langer T (2019). The Good and the bad of mitochondrial breakups. Trends Cell Biol..

[46] Windt G, O’Sullivan D, Everts B, Huang SC-C, Buck MD (2013). CD8 memory T cells have a bioenergetic advantage that underlies their rapid recall ability. Proc. Natl. Acad. Sci. USA.

[47] Balmer ML, Ma EH, Bantug GR, Grahlert J, Pfister S (2016). Memory CD8+ T Cells require increased concentrations of acetate induced by stress for optimal function. Immunity.

[48] Angelin A, Gil-de-Gomez L, Dahiya S, Jiao J, Guo L (2017). Foxp3 reprograms T cell metabolism to function in low-glucose, high-lactate environments. Cell Metab..

[49] Gerriets VA, Kishton RJ, Johnson MO, Cohen S, Siska PJ (2016). Foxp3 and Toll-like receptor signaling balance T_reg_ cell anabolic metabolism for suppression. Nat. Immunol..

[50] Patel SJ, Sanjana NE, Kishton RJ, Eidizadeh A, Vodnala SK (2017). Identification of essential genes for cancer immunotherapy. Nature.

[51] Rafiq S, Hackett CS, Brentjens RJ (2020). Engineering strategies to overcome the current roadblocks in CAR T cell therapy. Nat. Rev. Clin. Oncol..

