# Supplementary material for: Nanoparticle-Mediated Lipid Metabolic Reprogramming of T Cells in Tumor Microenvironments for Immunometabolic Therapy
Source: Nanomicro Lett. 2021 Jan 4;13:31. doi: 10.1007/s40820-020-00555-6 (PMC8006499; doi:10.1007/s40820-020-00555-6)
Supplement: Supplementary file 1 — Supplementary material 1 (PDF 1854 kb) [file 40820_2020_555_MOESM1_ESM.pdf]

Supporting Information for

Nanoparticle-Mediated Lipid Metabolic Reprogramming of T Cells in Tumor Microenvironments for Immunometabolic Therapy

Dongyoon Kim[1], Yina Wu[1], Qiaoyun Li[1], Yu-Kyoung Oh[1, *]

[1]College of Pharmacy and Research Institute of Pharmaceutical Sciences, Seoul National University, Seoul 08826, Republic of Korea

*Corresponding author. E-mail: ohyk@snu.ac.kr (Yu-Kyoung Oh)

Supplementary Figures

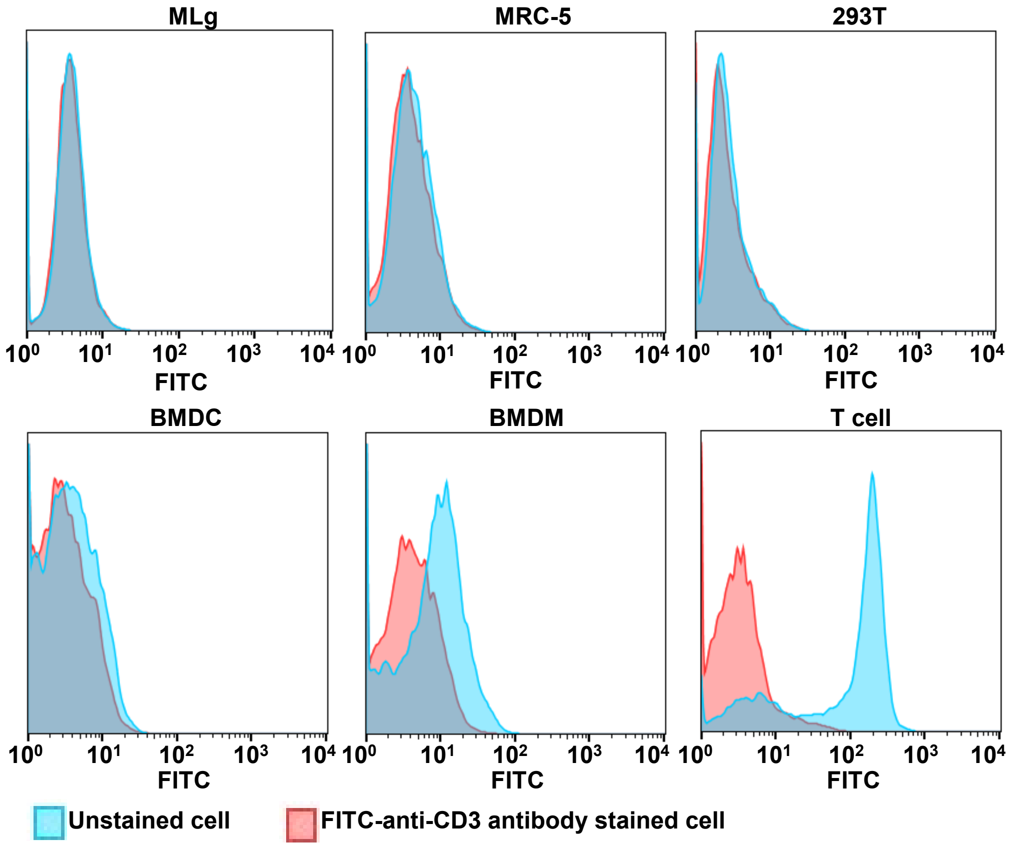

Fig. S1 CD3 expression levels on various cells. Flow cytometry was used to measure the expression levels of CD3 on six different cell types including murine lung fibroblast cell line MLg, human lung fibroblast cell line MRC-5, human embryonic kidney cell line 293T, BMDC, BMDM, and T cells

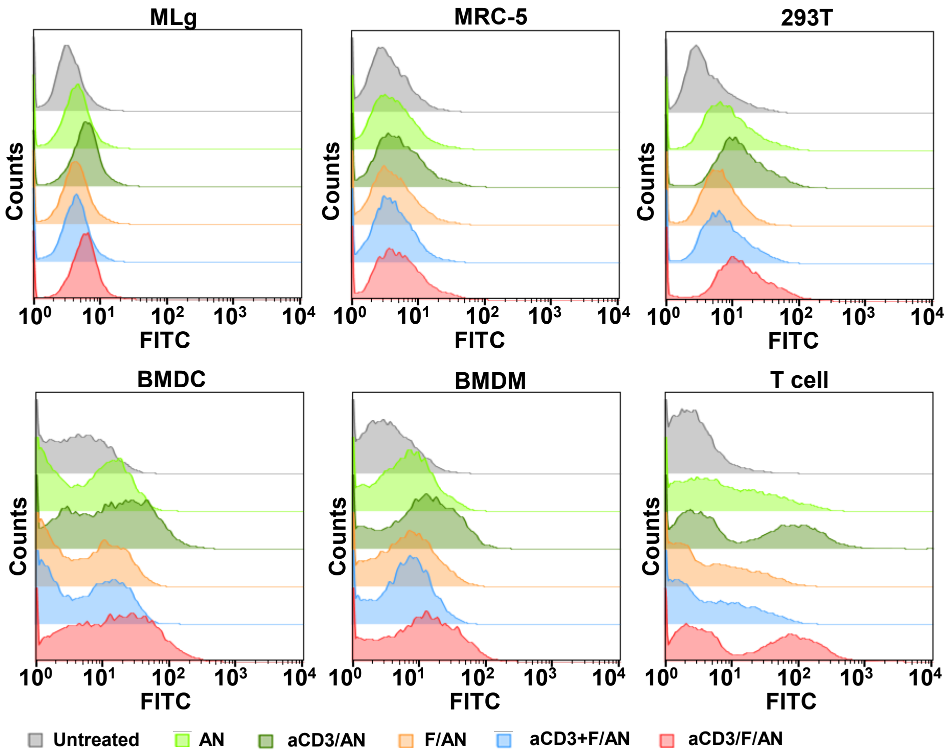

Fig. S2 Uptake of nanoparticles in various cells. MLg, MRC-5, 293T, BMDC, BMDM, and T cells were treated with various nanoparticles tagged with fluorescent marker. After 4 h, cellular uptake of nanoparticles was measured by flow cytometry

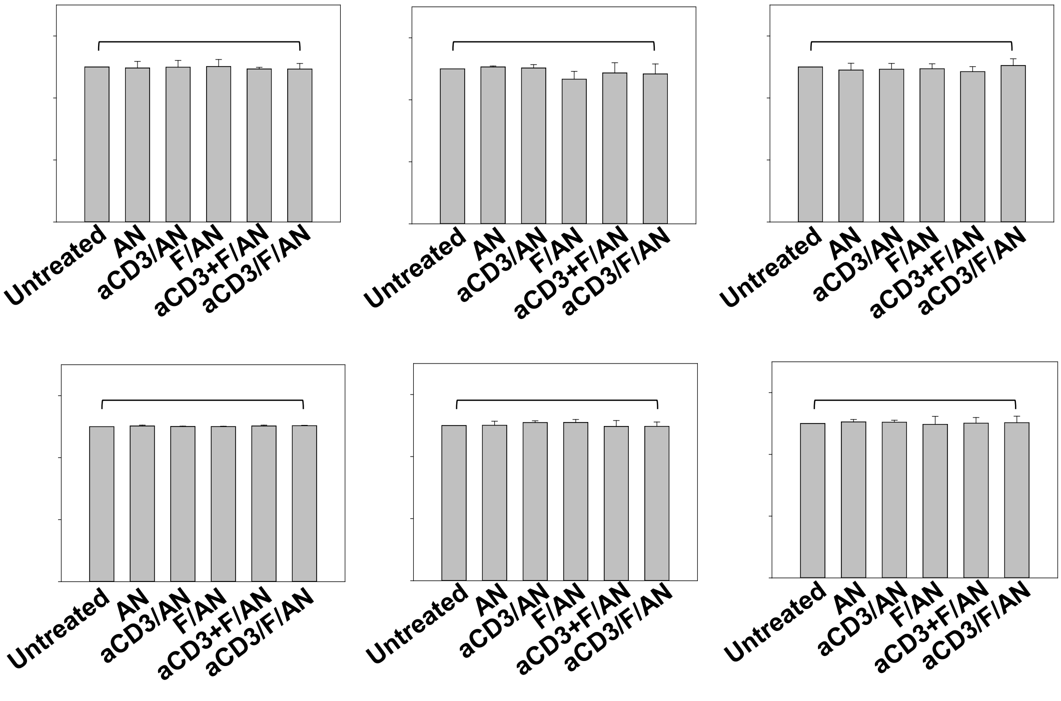

Fig. S3 Viability of cells treated with various nanoparticles. Viability of cell of nanoparticles on normal cells and T cell. MLg, MRC-5, 293T, BMDC, BMDM and T cells were treated with various nanoparticles. After 24 h, cell viability was measured by MTT assay for adherent cells and WST-1 assay for suspension T cell (n.s., not significant)

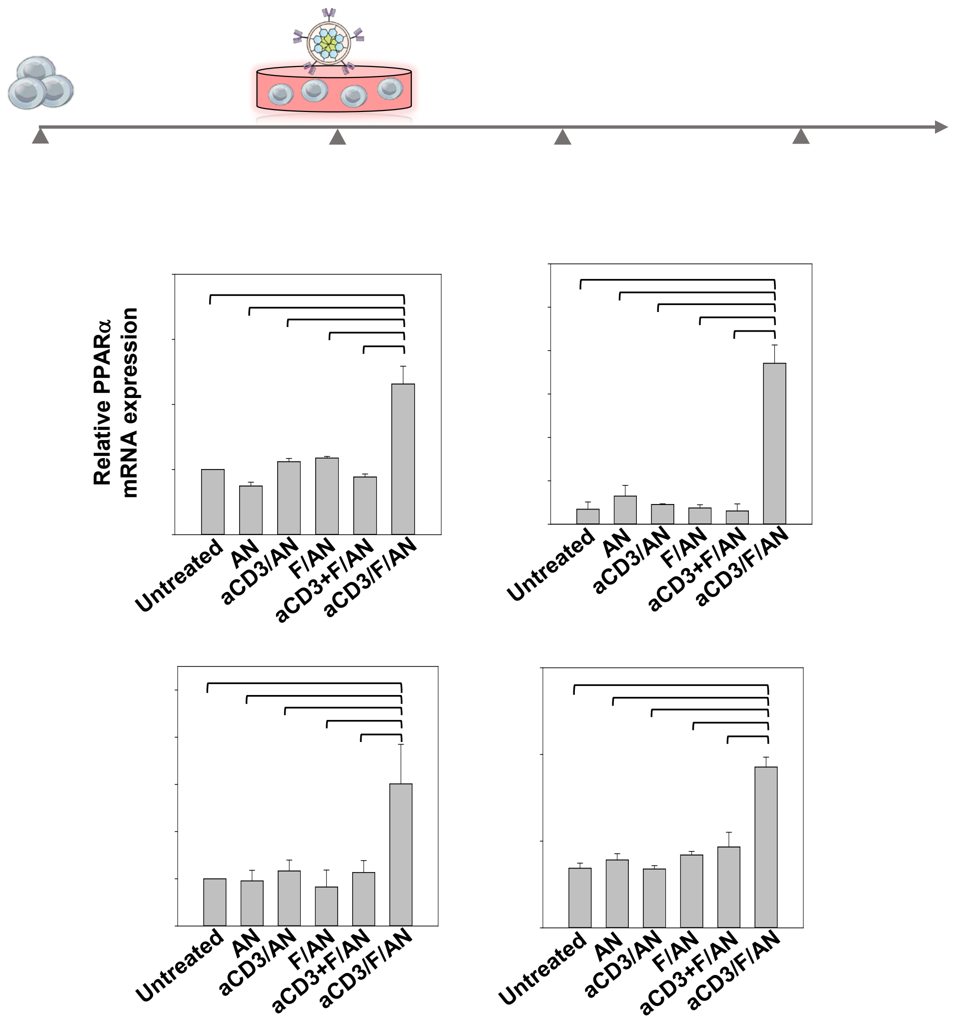

Fig. S4 mRNA expression levels of PPARα and CD36 in T cells. **a** Splenic T cells were treated with various nanoparticles, and the mRNA expression levels of PPARα and CD36 in CD3^{+} T cells was analyzed. **b** One day after various nanoparticle treatment, mRNA expression levels of PPARα in T cells were quantified by RT-PCR. **c** Two days after various nanoparticle treatment, protein expression levels of PPARα in T cells were measured by flow cytometry. **d** One day after various nanoparticle treatment, mRNA expression levels of CD36 in T cells were quantified by RT-PCR. **c** Two days after various nanoparticle treatment, protein expression levels of CD36 in T cells were measured by flow cytometry (***P<0.001)

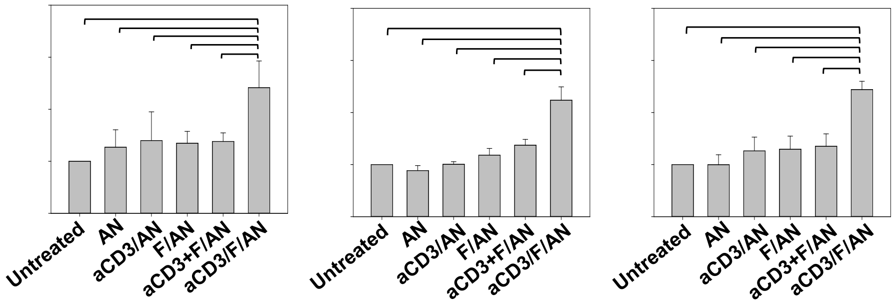

Fig. S5 Protein expression levels of CPT1B, LCAD and MCAD. Western blot bands of CPT1B, LCAD and MCAD were quantified by density analysis, and normalized to those of b-actin (*P<0.05, **P<0.01, ***P<0.001)

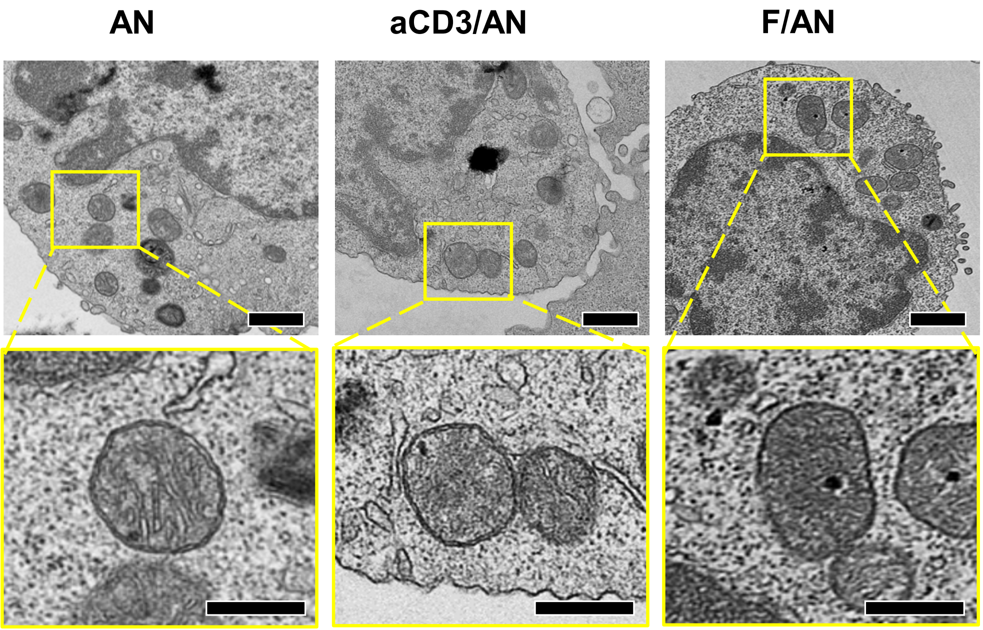

Fig. S6 TEM showing the morphology of mitochondria treated with AN, aCD3/AN, and F/AN. T cells were treated with various nanoparticles and incubated in low-glucose medium containing palmitate as a lipid source. Scale bars: 1 mm (upper panels) and 500 nm (lower panels)

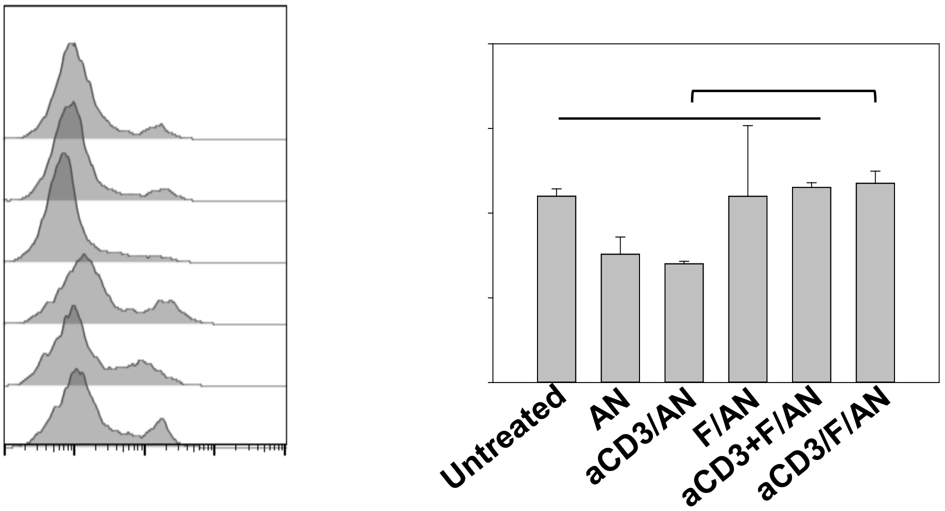

Fig. S7 Mitochondrial membrane potential in T cells under low-glucose conditions. T cells were treated with various nanoparticle preparations for 48 h and incubated in low-glucose medium. After 24 h, cells were harvested and incubated with a mitochondrial membrane potential-responsive fluorescent dye. The mitochondrial membrane potential was analyzed by flow cytometry (**a**) and the mean fluorescence intensity was quantified (**b**) (n.s.: not significant [P >0.05])

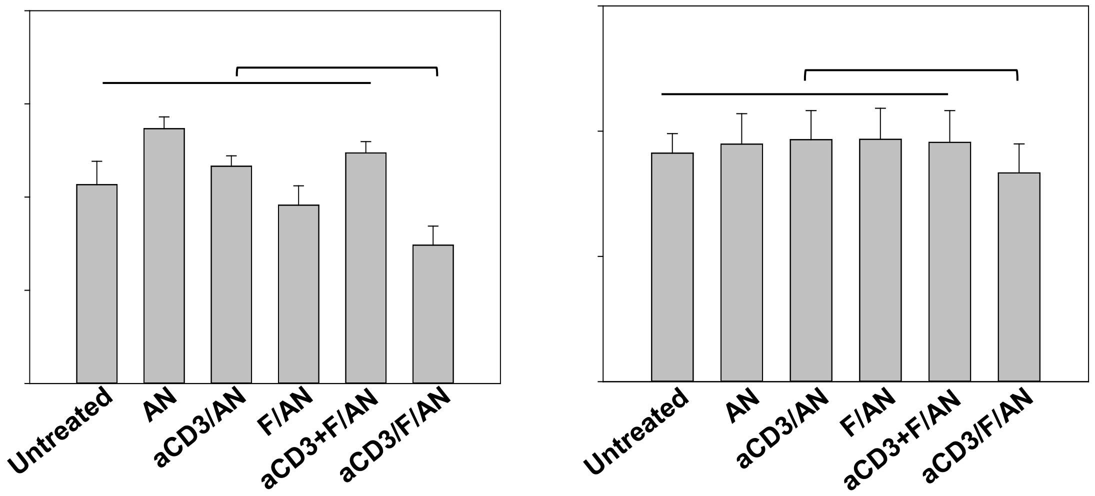

Fig. S8 Lipid metabolism and T cell survival under low-glucose conditions. T cells were treated with various nanoparticle preparations for 48 h and incubated in low-glucose medium. After 24 h, media were collected and levels of secreted β-hydroxybutyrate were analyzed (**a**). T cells were harvested and the annexin V^{-}/PI^{-} T cell population was quantified by flow cytometry (**b**) (n.s.: not significant [P >0.05])

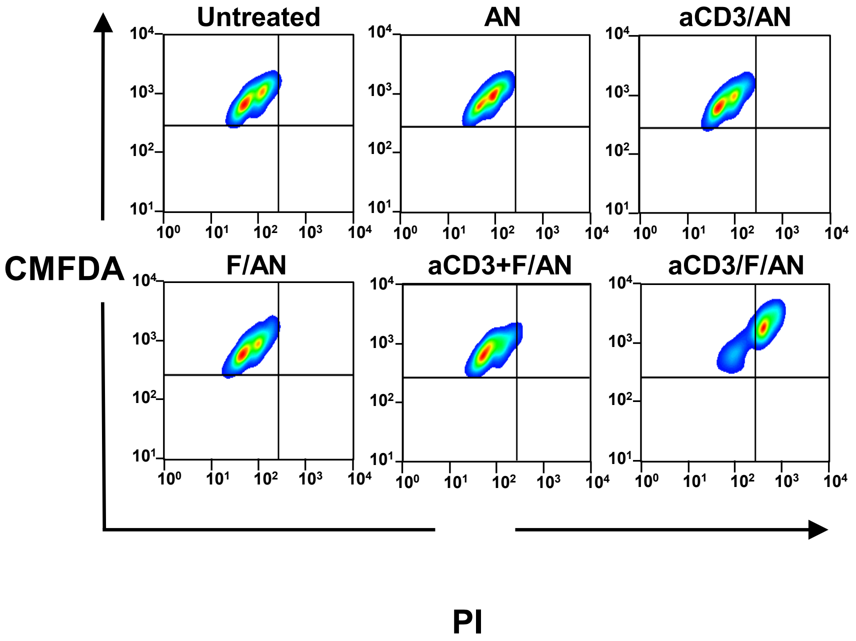

Fig. S9 In vitro cancer cell-killing activity of aCD3/F/AN-treated T cells. T cells were treated with various nanoparticle preparations for 48 h and coincubated with CMFDA fluorescent dye-stained B16F10 tumor cells in low-glucose medium containing palmitate as a lipid source. After 24 h, the $CMFDA^{+}/PI^{+}$ T cell population corresponding to dead tumor cells was analyzed by flow cytometry

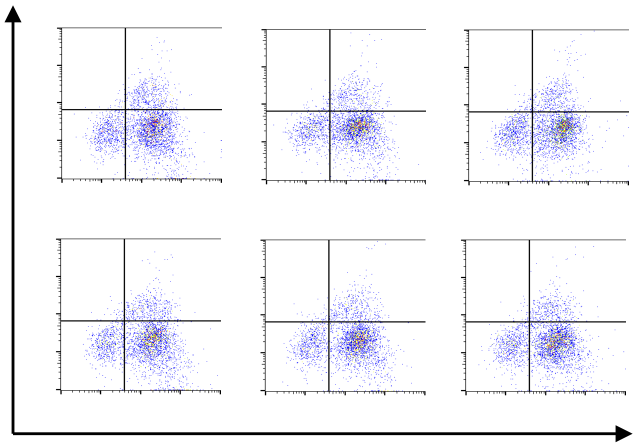

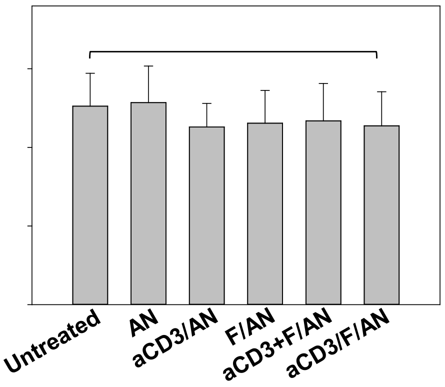

Fig. S10 FoxP3 expression of T cells treated with nanoparticles. **a, b** T cells were treated with various fenofibrate-containing nanoparticles. After 48 h, the levels of FoxP3 in T cells ($CD3^+/CD4^+/CD25^+/FoxP3^+$) were evaluated by flow cytometry (**a**), and quantified (**b**) (n.s., not significant)

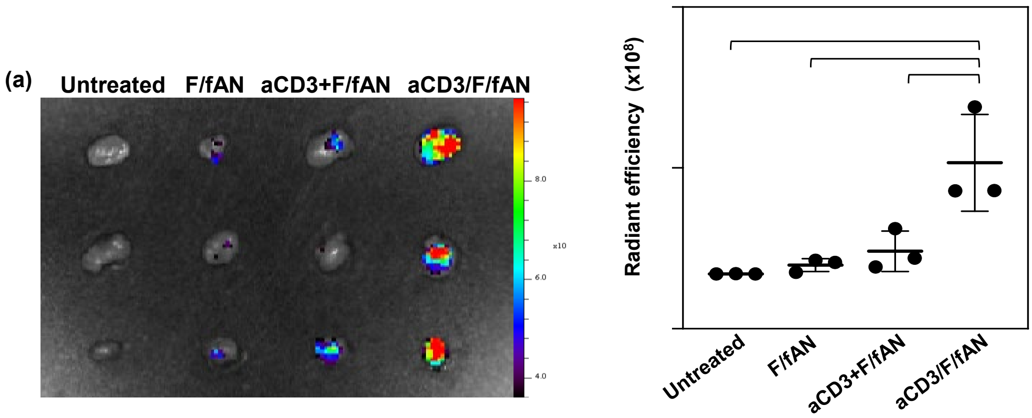

Fig. S11 In vivo tumor tissue retention of nanoparticles. FITC-labeled nanoparticles were intratumorally injected into B16F10 tumor-bearing mice. Three days after injection, tumor tissues were extracted, and the ex vivo fluorescence of tumor tissues was visualized (**a**) and quantified for each group (**b**) (**P<0.01)

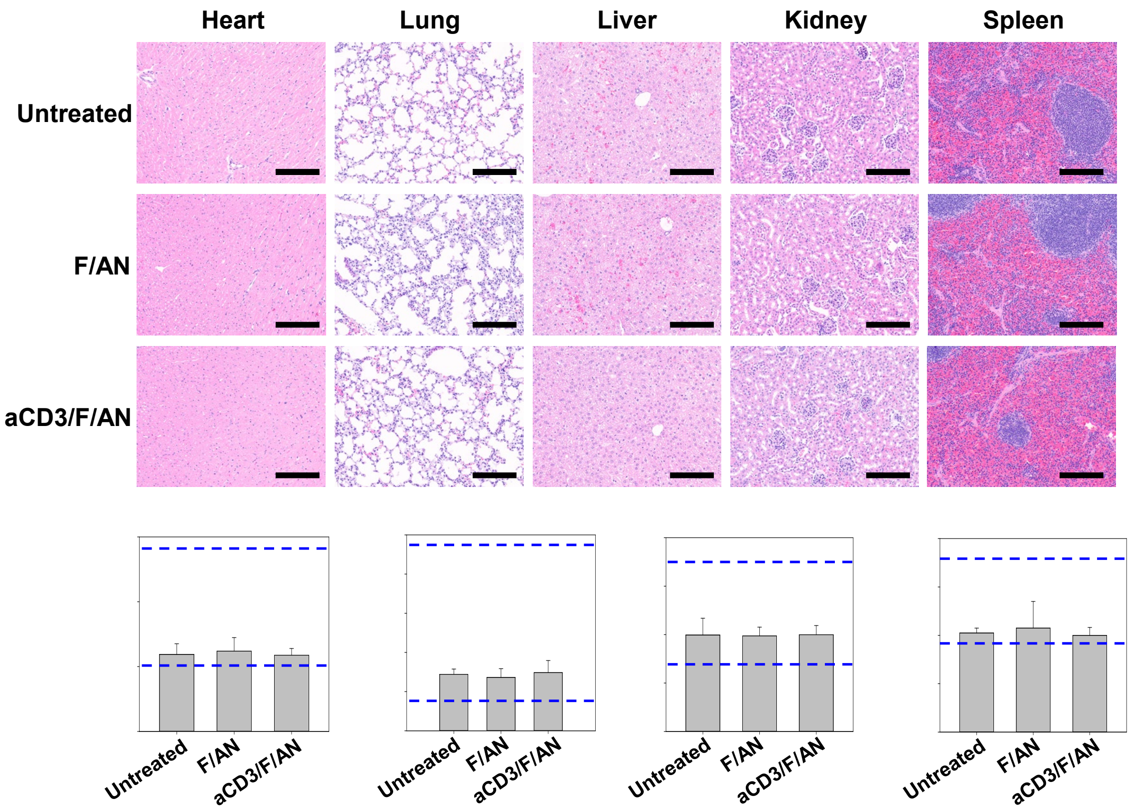

Fig. S12 Safety of aCD3/F/AN in normal mice. **a** Mice were subcutaneously injected with F/AN or aCD3/F/AN twice with two-day interval (n=5). Whole blood and major organs were extracted 2 days after the second injection. Major organ tissue toxicity of heart, lung, liver, kidney and spleen were evaluated by hematoxylin and eosin staining. Scale bar: 200 mm. **b** Biochemical parameters were evaluated for ALT, AST, BUN, and creatinine. Blue dotted line shows the normal ranges of each parameter. **c** Hematological parameters were evaluated

Table S1 List of primers used in RT-PCR

Gene	Forward	Reverse
PPARα	5'-AGCCCCATCTGTCCTCTCTCC-3’	5'-TCCAAGAGCTCTCCTCACCGATG-3'
CD36	5'-GGAGCCATCTTTGAGCCTTCA-3’	5'-GAACCAAACTGAGGAATGGATCT-3'
CPT1B	5'-CCTCCGAAAAGCACCAAAAA-3’	5'-GCTCCAGGGTTCAGAAAGTAC-3'
LCAD	5'-GGTGGAAAACGGAATGAAAGG-3’	5'-GGCAATCGGACATCTTGAAAG-3'
MCAD	5'-TGTTAATCGGTGAAGGAGCAG-3’	5'-TGTTAATCGGTGAAGGAGCAG-3'
GAPDH	5'-ATCACCATCTTCCAGGAGC-3’	5'-AGAGGGGCCATCCACAGTCTTC-3'
